# Mitigation of gas-induced damage in bipolar membranes for CO_2_ electrolysis

**DOI:** 10.1039/d5ta04879f

**Published:** 2025-09-12

**Authors:** Robert Fischer, Matthieu A. Dessiex, Lorenz Gubler, Sophia Haussener, Felix N. Büchi

**Affiliations:** a PSI Center for Energy and Environmental Sciences 5232 Villigen PSI Switzerland robert.fischer@esrf.fr felix.buechi@psi.ch; b Laboratory of Renewable Energy Science and Engineering, Ecole Polytechnique Fédérale de Lausanne (EPFL) 1015 Lausanne Switzerland

## Abstract

Bipolar membranes operated in forward bias are a promising platform for CO_2_ electrolysis, enabling alkaline cathode environments and pure-water feed while preventing CO_2_ crossover and salt precipitation. However, their deployment is limited by structural degradation under sustained operation. Here, we present a systematic investigation of degradation using X-ray tomographic microscopy, capturing the evolution of membrane delamination and anode catalyst layer damage as a function of current density and passed charge. Our results demonstrate that membrane delamination strongly depends on current density, while anode catalyst layer degradation scales with cumulative charge, highlighting distinct degradation pathways within the membrane-electrode assembly. To mitigate these effects, we engineer porous anion exchange layers to enhance CO_2_ back-diffusion and relieve interfacial gas pressure. Among several architectures, a *microporous* anion exchange layer fabricated *via* nanoparticle-ionomer spray coating shows the most effective suppression of both membrane delamination and anode catalyst layer damage, while achieving high current densities and improved faradaic efficiency for CO production. These findings establish gas transport engineering within the bipolar membranes as a critical design lever for achieving durable and efficient CO_2_ electrolysis.

## Abstract 2

Bipolar membranes operated in forward bias are a promising platform for CO_2_ electrolysis, enabling alkaline cathode environments and pure-water feed while preventing CO_2_ crossover and salt precipitation. A porous AEL prevents gas induced damages.

## Graphical abstract

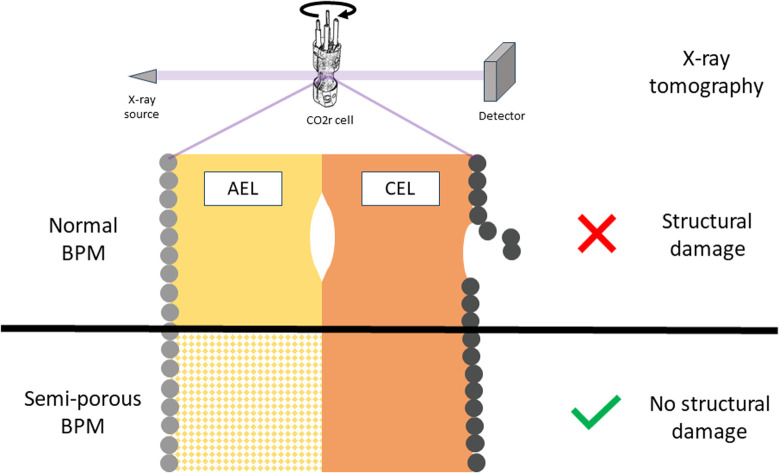

## 1 Introduction

The chemical reduction of captured CO_2_ by renewable energy to chemical feedstock is a desirable technology to replace fossil sources and decrease net CO_2_-emissions to the atmosphere. Different approaches have been proposed including CO_2_ hydrogenation or solar thermochemical,^1–5^ photocatalytic,^6–8^ electrocatalytic CO_2_ reduction^9–11^ and combinations thereof.^12,13^ Electrocatalytic CO_2_ reduction can be performed in a setup independent of environmental conditions and flexibly operated according to the demand and supply from the electricity grid. Among the different approaches to electrocatalytic CO_2_ reduction,^9–11^ a gas-fed zero-gap electrolyser design that utilizes an ion-conducting membrane and aims to produce carbon monoxide (CO) shows promise of high current density, high selectivity, and good product value.^14–16^

**Fig. 1:**
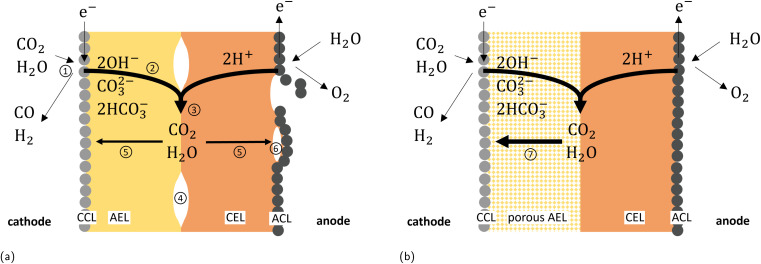
(a) Species pathways for forward bias BPM CO2ELY adapted from.^23^ 1: desired CO_2_ reduction to CO, and carbonate formation, 2: carbonate as anionic charge carriers, 3: ion recombination forming CO_2_ and water, 4: membrane delamination after sufficient CO_2_ pressure build-up, 5: CO_2_ backdiffusion and crossover, 6: CO_2_ gas formation above solubility limit damaging anode catalyst. (b) Situation with semi-porous BPM, with 7: enhanced backdiffusion of the recombined species towards the cathode.

The current state-of-the-art for zero-gap cells of CO_2_ electrolysis (CO2ELY) employs an anion exchange membrane (AEM) to provide the alkaline environment at the cathode that favors CO_2_ reduction over hydrogen evolution. However, AEM-CO2ELY fundamentally suffers from CO_2_ crossing to the anode in (bi)carbonate form and fast degradation by carbonate salt formation.^17–22^ The set-up with bipolar membranes (BPM) operated in forward bias solves both these issues. There, the anion and cation exchange layer (AEL, CEL) face the cathode and anode, respectively, which retains the favorable alkaline cathode conditions, alleviates CO_2_ crossover, and prevents salt formation by feeding the anode with pure-water instead of a salt solution, which could further give a decisive advantage for long term stability by maintaining a non-corrosive environment.^15,23–27^ While BPMs in forward bias have successfully been employed for CO2ELY, the concept still faces open challenges, including high ohmic resistance^26,28^ and even faster degradation than AEM-CO2ELY.^20,23,25,29^ The degradation of BPM-CO2ELY performance is a complex issue. It includes the loss of cations, which have a beneficial effect on the local environment of the electro-catalyst^18,21,22,30–32^ as well as structural damage to the membrane electrode assembly (MEA).^23,29^ For instance, we reported in a previous study^23^ gas-induced damage to the anode catalyst layer (CL) and membrane delamination due to the formation of CO_2_ at the BPM junction where the AEL and CEL are in contact. Fig. 1a illustrates species pathways and corresponding structural damage in forward-bias BPM-CO2ELY. Local CO_2_ production at the membrane interface is directly linked to current flow through ion recombination. While the damage to the anode catalyst layer (ACL) occurred at all operating conditions and appeared to depend on the cumulatively passed charge density, membrane delamination could only be observed for elevated current densities.^23^ We hypothesized that a balance of current-density-induced ion recombination and diffusive CO_2_ removal leads to a CO_2_ pressure buildup and eventual delamination at the AEL-CEL interface. Contrary to the dynamic equilibrium at the AEL-CEL interface, CO_2_ trapped in pockets between CEL and ACL is released only once by breaking the ACL (Fig. 1a), hypothetically explaining the charge-damage relation of the ACL.^23^

In this work, we systematically investigate the influence of charge and current density on structural degradation of bipolar membranes (BPMs) in CO_2_ electrolysis (CO2ELY), and assess the extent to which such degradation can be mitigated by modifying the anion exchange layer (AEL) of the BPM. Experimentally, the study is based on imaging, using the moderately invasive method of X-ray tomographic microscopy (XTM)^33^ to monitor mechanical processes that are inaccessible by the electrochemical and chromatographic methods employed for performance analysis and faradaic efficiency determination.^17,19,21,22,34,35^ However, while being used for two decades for fuel cells, XTM has only recently been employed in CO2ELY research.^23,36,37^ To address degradation driven by gaseous CO_2_ accumulation, we explore membrane designs that enhance CO_2_ transport toward the cathode.^29,38–40^ Introducing gas transport pathways through ’semi-porous’ BPMs (Fig. 1b) has been shown to reduce membrane delamination^29^ and improve CO_2_ utilization.^38^ Here, we evaluate how different porous AEL architectures within the BPM influence both degradation resistance and faradaic efficiency for CO production. BPM with thin AEL^26,41^ and applying a high differential pressure^41^ also show good and stable performance potentially indicating enhanced CO_2_ back-diffusion and membrane stabilization at currently achievable current densities of several 100 mA cm^−2^. We will discuss our results in the context of the fundamental transport mechanism and projected current densities beyond 1 A cm^−2^.

## 2 Materials and methods

### 2.1 Catalyst layer preparation

The electrodes are prepared similarly as in our previous studies:^23,25,42^ The cathode is a gas diffusion electrode (GDE). The cathode catalyst ink is composed of 92 wt% silver nanoparticles (APS 20–40 nm, Alfa Aesar, Schiltigheim, France) mixed in an anion-conducting ionomer (Piperion-A TP85, Versogen, Newark, Delaware, US). The ionomer was initially in resin form but was dissolved at 2.5 wt% in ethanol. In preparation for spray coating, the nanoparticle and ionomer were further dispersed in 50–50 vol% ultrapure water and isopropanol. The cathode catalyst ink was then sprayed with an Ag loading of 2 mg cm^−2^ onto the *microporous* layer (MPL) of a carbon paper GDL (H23C8, Freudenberg, Weinheim, Germany). The anode catalyst ink is composed of 90 wt% IrO_2_–TiO_2_ nanoparticles (Elyst Ir75 0480, Umicore, Brussels, Belgium) in a proton-conducting ionomer (5 wt% Nafion dispersion in aliphatic alcohol/water, EW = 1100 g mol^−1^, DuPont, purchased from Sigma-Aldrich). For spraying, the IrO_2_ nanoparticles and ionomer were further dispersed in 80–20 vol% ultrapure water and isopropanol. The anode catalyst ink was sprayed either on the cation exchange side of a *commercial* BPM (Fumasep FBM, Fumatech) or on a Nafion 115 membrane as a cation exchange layer (CEL) to form a catalyst-coated membrane (CCM). The target loading is 2.5 mg_IrO_2__ cm^−2^. The loading was measured by simultaneous spraying on a thin PTFE sheet.

### 2.2 Bipolar membranes

In total, five different bipolar membranes (BPM) were tested. The *commercial* Fumasep BPM (Fumatech, Germany) was the base case. To avoid batch-to-batch variation, we used the same membrane batch throughout this study and the previous ones.^23,42^ The other four membranes were different BPMs made in-house, three of them with a porous AEL. The Nafion CCM serves as CEL for all four in-house BPMs. In three of those, FAA-3-50 (Fumatech) was employed as AEL in three variations: *custom*, *macroporous* and *mesoporous*. *Custom* is FAA-3-50 without modification, the *macroporous* AEL was manually perforated with a hypodermic needle (0.65 mm) in a 1.25 mm square pattern guided by a template. For the *mesoporous* AEL with smaller holes in higher density, a spring-loaded insect pin tool (size 000, Fine Science Tools)^43^ was mounted to a Computer Numerical Control (CNC) machine and driven in a programmed route to produce a hexagonal (*a* = 0.25 mm, *b* = 0.5 mm) grid. At every point, the needle was driven 3 mm into the membrane. Contrary to the hypodermic needle for the *macroporous* perforation, the insect pin tool only produces about 40 μm long linear tears. No material is removed, other than for the *mesoporous* AEL. The AELs were then soaked for 6 h in 0.5 M KOH solution, for 72 h in 0.5 M K_2_CO_3_ and rinsed thoroughly in de-ionized water between steps.

As a final modification, a *microporous* AEL was deposited by spraying a suspension of 90% wt TiO_2_-nanoparticles and 10%wt Piperion-A ionomer. The ionomer was initially in resin form and was dissolved at 2.5 wt% in ethanol. For the spraying ink, the nanoparticles and the ionomer were further dispersed in 50–50 vol% ultrapure water and isopropanol. The layer was spray-coated onto the Nafion 115 for the laboratory scale cell and onto the cathode GDE for the miniature cell for imaging due to the difficulty of having a double-sided CCM on such a small active area. The target loading was 3.5 mg cm^−2^. Microscopic images of the porous AELs are given in SI Fig. S1. Fig. S1e shows that the porous layer is about 30 μm thick. With the given loading, a porosity of about 65% is calculated. The SEM images were recorded with a Carl Zeiss Ultra55 scanning electron microscope at an electron high tension of 5.50 kV. Prior to SEM, a 5 nm Au-layer was sputtered onto the sample to improve its electron conductivity with a Leica EM ACE200 vacuum coater. For cross-sectional observations, liquid nitrogen was used to freeze the membrane before breaking it.

Except for the *commercial* and the *microporous* BPM, the CEL and AEL are placed on top of each other to produce the BPM during cell assembly.

### 2.3 Electrolyzer cell

#### 2.3.1 Principal cell design

We used two differently sized electrolyzer cells, optimized for imaging and testing electrochemical performance, respectively. Our miniature electrolysis cell (active area 10 mm^2^) with two parallel channels in the flow field (FF)^23,42^ was employed to combine electrochemical operation with XTM. Faradaic efficiency could not be evaluated with the miniature cell, and was therefore measured on a reliable laboratory scale cell (active area: 4 cm^2^)^25^ with inline gas analysis to ensure the applicability of the cell performance achieved by the miniature cell for imaging and avoiding size effects. The cell features a parallel FF configuration with the geometry: 0.8 mm (height) × 0.8 mm (width) × 0.8 mm (rib pitch).^25^ When specified, we change the cathode FF to a serpentine design with the geometry 0.4 mm (height) × 0.6 mm (width) × 0.6 mm (rib pitch). The cell components, other than the bipolar plates and the peripheral connections, are the same if not mentioned otherwise. The membrane electrode assembly (MEA) consists of the above-described cathode GDE, CCM (BPM with anode CL) and a Ti porous transport layer (GDL10, Bekaert, Belgium, PTL). In our previous work^23^ with the *commercial* BPM, a carbon paper gas diffusion layer (GDL, SGL 29 AA) was used as PTL at the liquid water-fed anode. Carbon has a lower X-ray attenuation than titanium and reduces metal artifacts in the images. We tested 11 samples with the *commercial* BPM at various operating conditions with the GDL-assembly in the miniature cell to test the synchrotron experiments for reproducibility, while the remaining samples use the Ti-PTL typical for electrolyzers to exclude the influence of the more flexible carbon GDL. After assembly, the miniature cell was compressed with two flanges to ensure gas and water tightness. The laboratory scale cell features a spring mechanism to control the contact pressure, which was aimed to be 20 bar.

#### 2.3.2 Operation of the miniature cell for imaging

After cell assembly, one tomographic scan was taken to obtain the state at the beginning of life (BOL). The sample, still mounted on the sample holder, was then transferred to a fume hood and connected to the potentiostat, gas and water flows, which were set up in the same way as in^23^: CO_2_ from a pressurized bottle reduced to 1 bar, passes by a mass flow controller set to 7.5 mL min^−1^ through a room temperature bubbler before entering the cell (estimated mean flow velocity in the FF of 0.16 m s^−1^). Millipore water was recirculated through the anode FF from a bottle placed in a bath set to 55 °C, giving a cell temperature of 45 °C due to thermal losses in the tubings and the cell. The water was replaced for every sample to avoid potential contamination. At the start of the experiment, a preconditioning protocol was applied consisting of sequential voltage holds: 2.00 V, 2.25 V, 2.50 V, and 2.75 V for 3 minutes each, followed by 3.00 V, 3.50 V, and 3.75 V for 1 minute each. This protocol was automatically interrupted once the respective target current density had been reached to proceed to the actual operation protocol at constant current density. Then, the sample was operated for a fixed amount of passed charge (180 As cm^−2^, *e.g.* 100 mA cm^−2^ 30 min) while recording the total cell voltage. The operation time was adjusted for different current densities to achieve the same amount of passed charge. The cell was disconnected and transferred (<5 min) to the XTM scanner to start the acquisition of a tomographic scan (1st scan). Immediately after acquiring the scan, the cell was transferred back to the fume hood, connected and operated again for the same charge and current. Another scan was taken after the end of operation/life (EOL). In several cases, the assembled cells could not achieve the planned current density in galvanostatic operation. Those cells were still tested in potentiostatic operation (constant voltage) and investigated based on the maximal observed current density. Those same cells often could not be at all operated after the 1st scan, which in these cases corresponded to the EOL.

#### 2.3.3 Operation of the laboratory scale cell

The cell temperature was controlled by electric heating and a thermocouple. The cell temperature was set to 40 °C. The cathode feed was set to be 27 mL_n_ min^−1^ CO_2_ and 3 mL_n_ min^−1^ Ar, leading to an estimated mean flow velocity of 0.065 m s^−1^ for the parallel flow field, and 2.083 m s^−1^ for the serpentine flow field. If not specified differently, the cell pressure was set to 1.05 bar. For the two non-porous BPM (*commercial* and *custom*), the cathode feed was humidified to 50% relative humidity by passing the flow through a bubbler. The heated lines prevented any condensation from reaching the cell. The cathode outlet gas passes first through a humidity sensor (Vaisala HUMICAP 180RC Humidity and Temperature Transmitter HMT310), externally heated to 70 °C, before a Peltier cooler trapped and removed water from the gas stream. Finally, an in-line mass spectrometer (MS, Pfeiffer Omnistar GSD 320) was utilized for gas analysis.^25^ MS calibration followed the method proposed by Binniger *et al.*^44^ When no current is applied, the MS might still detect a signal for CO or H_2_, consequently the measured signal was calibrated for this spurious background. The anode feed was pure water, re-circulated, and passed through physical and ionic filters. The loop was heated to 40 °C. A water pump produces a flow of 25 mL min^−1^. The electric inputs are controlled *via* a potentiostat (Biologic SP-300, Booster 10A). After setting the cell and flow conditions, the cell was exposed to the flows for at least 1 hour. Then low constant current density (10 mA cm^−2^) was applied for 1 h. 10 fast constant potential steps were applied from 1.6 to 3.4 V holding the potential for 3 min each, then 4 longer constant potential steps were applied at 2.2 V, 2.6 V, 3.0 V, and 3.4 V for 20 min each. 3.4 V was held for an additional 3 h 40 min as the period of interest. Finally, the fast, constant potential steps from 1.6 V to 3.4 V (3 min holds) were repeated. The last minute of every constant potential step was dedicated to impedance measurement, where we recorded impedance from 200 kHz to 200 mHz, 6 points per decade, 10 mV sinusoidal amplitude, 0.1 period before each frequency, and an average of 2 measures per frequency. During the 3 h 40 min hold, the impedance was measured every 10 min. The faradaic efficiencies and partial current densities were calculated from the relative product-to-Argon measured in the mass spectrometer.

### 2.4 X-ray tomographic microscopy

X-ray tomographic microscopy (XTM) was performed on a tube X-ray cone beam labCT scanner (Phoenix nanotom, General Electric, Germany). The X-ray tube settings were 80 kV and 230 μA. A 0.1 mm thick copper sheet was placed on the X-ray source to filter out low energy photons and reduce metal artifacts in the images.^23^

The sample was placed at a distance of 12 mm to the X-ray source while the flat panel detector had a distance of 400 mm to the source capturing the entire width of the cell in the field of view and resulting in a voxel size of 3 μm. 1000 radiographic projections were recorded equally spaced over one full rotation of 360°. Each projection is the average of three frames at the same angle with an exposure time of 0.5 s each. The acquisition of one frame was skipped while the stage is moving to the next angular position. One tomographic scan took therefore, with some acquisition overhead, about 35 min. Tomographic reconstruction was performed using the implementation of the Feldkamp-algorithm for cone beams in the *commercial* software datos| rec (Waygate Technologies, Baker Hughes, USA). The result is a three dimensional image where each voxel has a grayvalue related to the material density. The absence of so called movement artifacts that would appear in the reconstruction if the sample deforms between angular projections, indicate that no relaxation of the membrane occurs during the acquisition.

### 2.5 Image analysis

#### 2.5.1 Basic image pre-processing

After reconstruction, several image processing steps are required to extract the perforated area of the catalyst layer and display potential membrane delamination. Since the mounted sample was not perfectly aligned with the three spatial directions of the three-dimensional image data, the sample was virtually aligned by subsequent manual rotation around the three axes. The edges of the bipolar plates were used as alignment reference and bicubic interpolation was employed to update the voxel grayvalues of the transformed image. The image data was then cropped to a volumetric region of interest (100 × 700 × 1300 voxel; 3 mm × 2.1 mm × 3.9 mm) deep and wide enough to include both catalyst layers and the active area. The 1st scan and EOL images were aligned in 3D to the BOL image to compensate for sample positioning using rigid-body registration with the center of the Ti-PTL as reference mask (simpleITK). The samples with carbon GDL could not be registered due to a lack of contrast. The registered images served as basis for the following analysis regarding membrane delamination and ACL damage. Image processing was implemented as *custom* Python code using the standard libraries SciPy, numpy and scikit-image.

#### 2.5.2 Membrane delamination

**Fig. 2:**
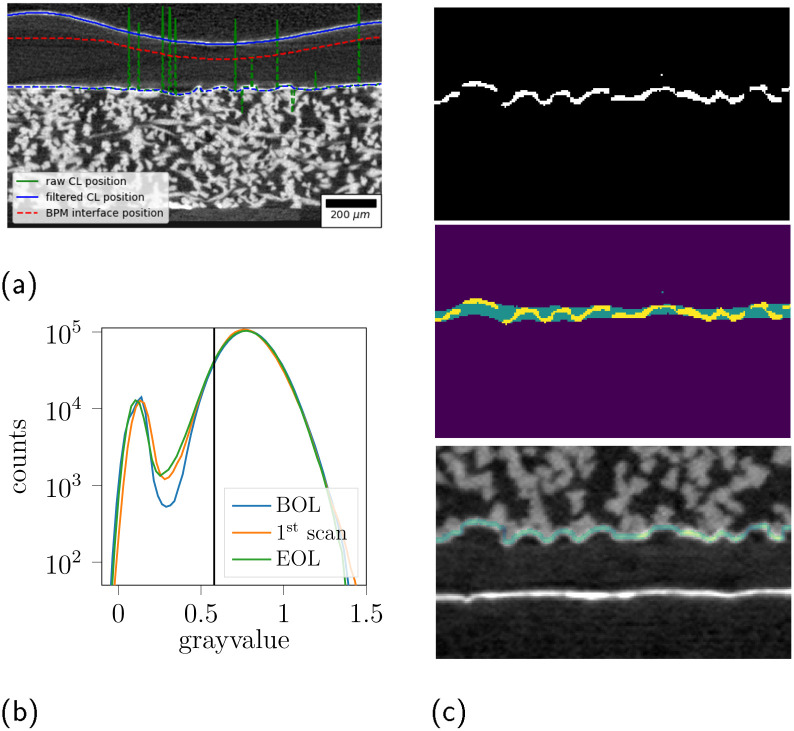
Illustration of image processing. (a) Cross-section of MEA, the raw CL positions (green) are filtered (blue) to give a isodistance cut at the AEL-CEL interface. (b) grayscale distribution within CL trace for different stages, left of the vertical bar are grayvalues not belonging to CL. (c) Illustration of trace extraction; top: raw CL segmentation by ML, middle: search band, bottom: overlay of expanded CL spine (=trace) on original grayscale image.

The limited image quality of the labCT compared to the synchrotron data,^23^ especially the more pronounced metal artifacts due to the cone beam, did not allow a segmentation of the membrane cavities. We opted for a qualitative analysis of the membrane state. We extracted an isodistance cut through the bipolar membrane to account for a deformed and non-aligned membrane. In the case of the *commercial* BPM, the isodistance was chosen at the center between the CL layers. For the other BPMs, the isodistance was 180 μm from the anode CL, which corresponds to the thickness of the Nafion membrane. The procedure is illustrated in Fig. 2a. Practically, the CL positions were extracted as grayscale gradient peaks in the image data, which were then corrected for image noise and holes developing in the anode CL by filtering and smoothing (Fig. 2a).

#### 2.5.3 Anode catalyst layer analysis

There is no clear separation in grayvalues between CL and CL-holes due to the limitation in signal noise, spatial resolution and metal artifacts. Especially cumbersome is the “catalyst shining” artifact caused by a combination of interaction processes of X-rays with thin, high absorbing metal layers.^42^ This “catalyst shining” artifact leads to an increase of voxel grayvalues in the proximity letting the CL appear thicker than its physical value and making CL hole detection difficult. The anode catalyst layer (ACL) was therefore analyzed in a multi-step procedure starting from the registered images. The procedure is illustrated in Fig. 2b and c and is described in the supplementary material. Effectively, we extract a ACL trace of the expected physical thickness (3 px = 9 μm) that bridges potential holes and segmentation mistakes in the ACL. We then extract a projection of the ACL as the through-plan mean grayvalue within the trace. By plotting the grayvalues histograms of the voxels within the trace, we can recognize a diverging point between the scans that indicates a suitable threshold (0.58) to distinguish voxels belonging to the ACL and to holes in the projection (Fig. 2b, and S32).

## 3 Results

In this section, we analyze the structural and electrochemical performance of different bipolar membrane (BPM) configurations for CO_2_ electrolysis. We begin by comparing the membrane architectures and physical characteristics. We then assess the evolution of degradation phenomena under operation and evaluate the impact of membrane design on faradaic efficiency and current stability.

### 3.1 Structural characteristics of bipolar membrane variants

Five BPM configurations were tested and are summarized in Table 1. All variants consist of a cation exchange layer (CEL) and an anion exchange layer (AEL), with systematic differences in material composition, thickness, and AEL porosity. The *commercial* BPM features a thin cast ionomer CEL and a dense AEL. While the exact composition is proprietary, thickness measurements from X-ray tomographic microscopy (XTM) indicate a total membrane thickness of approximately 88 μm. The *custom* BPM uses Nafion 115 as the CEL and an unmodified FAA-3-50 AEL, resulting in a total thickness close to 230 μm. Three additional BPMs were fabricated with porous AELs. The *macroporous* and *mesoporous* variants are based on the same base materials as the *custom* BPM, with perforation patterns introduced to modify porosity and gas permeability. The *microporous* BPM features a spray-coated AEL composed of TiO_2_ nanoparticles and anion-conducting ionomer, forming a 30 μm thick layer with a porosity of approximately 65%, as estimated from the loading and thickness. The CEL in all non-*commercial* BPMs is Nafion 115. Visual representations of the membrane cross-sections and perforation patterns are provided in Fig. S1. The porous AEL variants differ in porosity and the spatial distribution and characteristic size of the pores, ranging from isolated perforations (*macroporous*, *mesoporous*) to a continuous nanoporous network (*microporous*). In the subsequent sections, these membrane configurations serve as the basis for evaluating degradation behavior and electrochemical performance under CO_2_ electrolysis conditions.

**Table 1:** Overview of key characteristics of the BPMs. The exact composition of the *commercial* BPM is unknown. Membrane thickness was measured by XTM

Parameter	*Commercial*	*Custom*	*Macroporous*	*Mesoporous*	*Microporous*
Icon	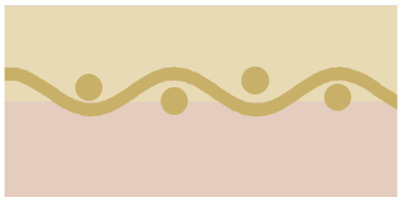	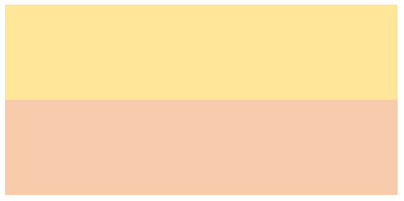	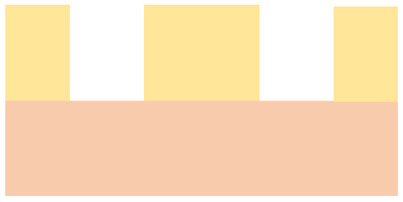	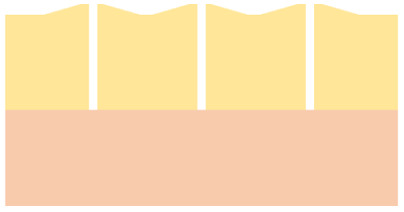	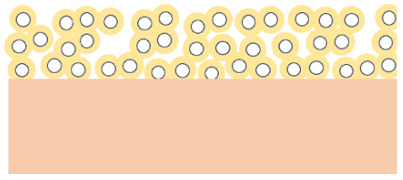
CEL	Cast ionomer (suspected)	Nafion 115	Nafion 115	Nafion 115	Nafion 115
CEL thickness	39 μm (ref. 42)	≈180 μm	≈180 μm	≈180 μm	≈180 μm
AEL	FAA-3-PK-75 (suspected)	FAA-3-50	FAA-3-50	FAA-3-50	Sprayed ink
AEL thickness	49 μm (ref. 42)	≈50 μm	≈50 μm	≈50 μm	≈30 μm
AEL porosity	0%	0%	≈21%	<1%	≈65%
Mean pore radius			0.325 mm (needle radius)	<10 μm (estimated)	400 nm (ref. 45) (estimated)

### 3.2 Membrane delamination

**Fig. 3:**
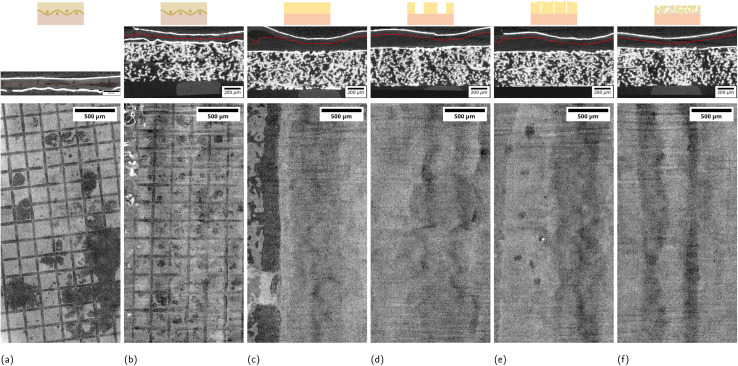
Top row: example cross-section indicating the isodistance cut as dashed line. Bottom row: EOL isodistance images of bipolar membrane after operation for 1 h at 100 mA cm^−2^ for the cases (a) *commercial* BPM with carbon GDL at anode, (b) *commercial* BPM with Ti-PTL at anode, (c) *custom*, (d) *macroporous*, (e) *mesoporous*, (f) *microporous*. Dark gray areas correspond to membrane delamination. The grid pattern in (a and b) are due to the reinforcement mesh. Light to dark gray vertical bands in (b–f) are caused by streak artifacts originating from the cathode CL bends.

Our experimental approach aims to decouple the influence of current density and passed charge on membrane delamination. Three XTM scans were taken: at the beginning of life (BOL), after an intermediate operation period (1st scan) and at the end of life (EOL) with a fixed amount of passed charge in-between. Fig. 3 shows virtual cuts through the BPM and illustrates the membrane state at EOL after operation at 100 mA cm^−2^ for an example of every BPM variant. All other membrane images are found in the SI (Fig. S3–S31). The dense AEL-variants (*commercial*, *custom*) show substantial delamination. The fiber reinforcement in the *commercial* variant constricts the delamination locally (Fig. 3a and b), while delamination occurs along the entirety of one channel in the *custom* variant (Fig. 3c). The BPM with porous AEL (*macro*-, *meso*-, *microporous*) show no membrane delamination (Fig. 3d–f). Table 2 gives a simplified overview of the observed membrane damage for all tested current densities, but fixed charge intervals. Inspecting Table 2 in detail, we recognize a minimal required current density to cause delamination in the *commercial* case. Cells with the *commercial* BPM-variant operated at 50 mA cm^−2^ or lower show no or only weak delamination and substantial delamination can only be observed for current densities of 100 mA cm^−2^ or higher. The extent of delamination in the *commercial* variant at 100 mA cm^−2^ also decreases qualitatively when employing a stiff Ti-PTL at the anode instead of the more flexible carbon fiber GDL. The *custom* BPM, however, shows substantial delamination for all operating conditions. We observe in both non-porous cases that delamination has already nearly fully developed after the first operation interval at fixed passed charges, as is visible in the 1st scan, followed by comparatively small changes until EOL (Table 2 and SI Fig. S3–S31). Once formed delaminations also do not disappear again. The *mesoporous* and *microporous* BPM-variants do not suffer from delamination even at elevated current densities (>200 mA cm^−2^). While the *macroporous* BPM prevents additional delamination, the area of the removed AEL material already corresponds to the delaminated area in the *commercial* and *custom* cases (SI Fig. S3–S31).

**Table 2:** Matrix of membrane delamination for BPM-types and operating conditions.^a^ All corresponding images are found in the SI as Fig. S3–S31

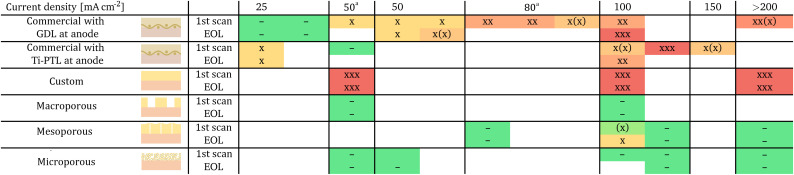

a Maximal measured current density for potentiostatic operation. Legend: – no delamination, x some delamination, xx big delamination, xxx large areal delamination, (x) between delamination stages. The two lines give the operation stages 1st and EOL. All membranes are intact at BOL multiple columns per current density are repeats.

### 3.3 Anode catalyst layer damage

**Fig. 4:**
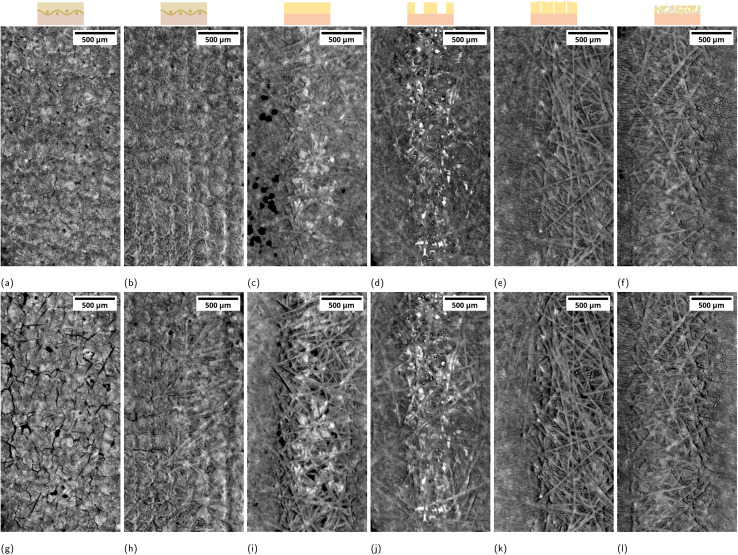
ACL projections after operation for 1 h at 100 mA cm^−2^ for the cases (a and g) *commercial* BPM with carbon GDL at anode, (b and h) *commercial* BPM with Ti-PTL at anode, (c and i) *custom*, (d and j) *macroporous*, (e and k) *mesoporous*, (f and l) *microporous*. Top row (a–f) BOL, bottom row (g–l) EOL. (c) Shows some uncorrectable processing artifacts in the shape of black blobs.

**Fig. 5:**
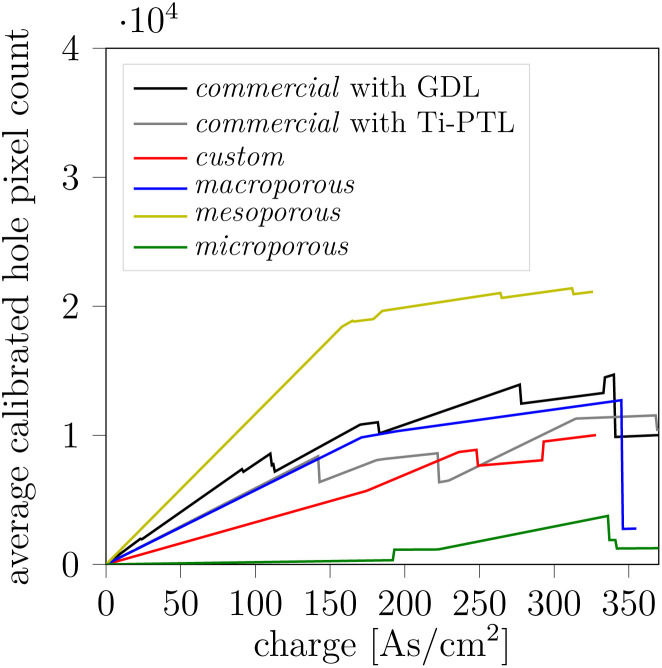
ACL damage as hole pixel count *versus* charge. Average per BPM-variant after calibration to pre-existing damage and interpolation to account for differing exact experimental charge values when taking a XTM scan. Individual relations in the SI Fig. S33.

The analysis of the anode catalyst layer (ACL) morphology evolution proved to be challenging and requires careful consideration, particularly due to the “catalyst shining” artifact and the indirect method used to extract a threshold for ACL holes. Fig. 4 shows the ACL at BOL and EOL as image projections for the same example samples as in Fig. 3 operated at 100 mA cm^−2^. We refer to the materials section for a detailed explanation of how the images were obtained. In general, dark spots indicate ACL holes, including holes both present at BOL and formed during operation. We notice fiber impression marks for the cases where a Ti-PTL was employed at the anode. These marks become more pronounced in the EOL scans (Fig. 4h–l) due to the impression of the ACL into the PTL by membrane swelling (Fig. 3).^42^ These fiber imprints can be mostly attributed to image and processing artifacts due to deformed interfaces of highly X-ray-absorbing materials. Fig. 4 looks qualitatively comparable to the ACL damage we had observed at our synchrotron campaign with the same cell setup.^23^ Holes and cracks can already be present at BOL (Fig. 4). The cumulated dark area is then used as a quantitative metric for ACL damage and is plotted in Fig. 5. We observe a wide spread of initial hole area at BOL introduced during the catalyst ink drying and cell assembly (Fig. S33a). We want, therefore, to focus on observable changes during and after operation. Within each BPM-variant, the ACL damage correlates with the passed charge. Fig. 5 shows the average curve of damage *versus* charge for each BPM-variant. The staggered curve is a consequence of the data processing. Due to practical reasons, including the preconditioning voltage ramp-up, the measured charge for the XTM-scans is not exactly the same for all samples. To compensate for this variation, the measurement points are linearly interpolated and resampled to 1 As cm^−2^. The average is then taken from the resampled curves and plotted in Fig. 5. Most BPM-variants show a similar, apparently linear, charge-damage relation. Exceptions are the *mesoporous* BPM with much higher damage and the *microporous* BPM with very low damage development. The quantitative analysis aligns well with the qualitative observation in Fig. 4 with low ACL damage for the *microporous* variant. Fig. 4l also points towards a different ACL cracking pattern in the *microporous* BPM (Fig. 4g–k) compared to the other variants which have break-out holes.

### 3.4 Faradaic efficiency and electrochemical performance

**Fig. 6:**
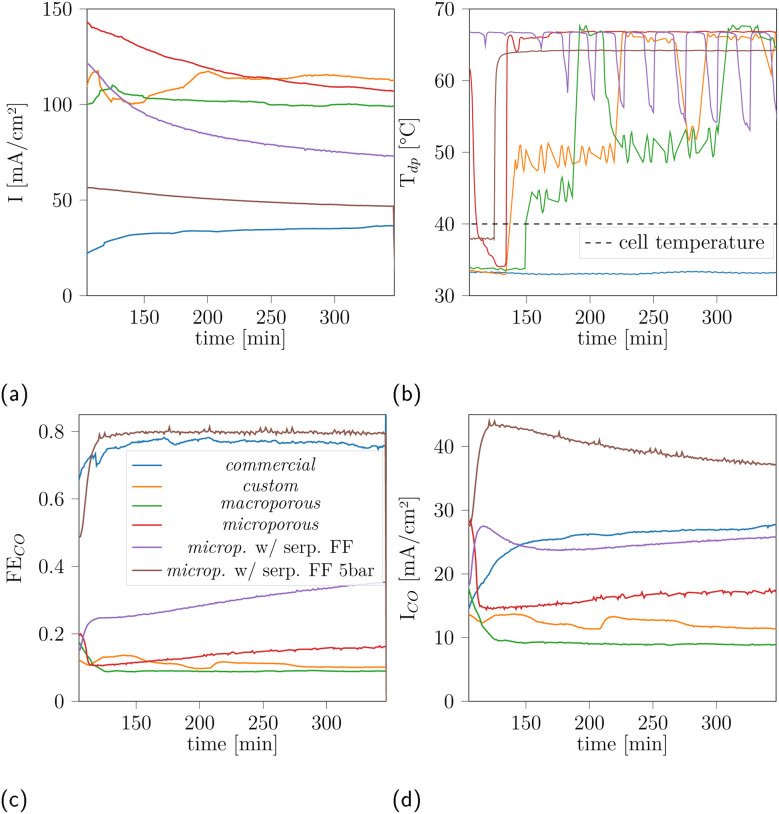
(a) Total current density over time for the potentiostatic period at 3.4 V, (b) outlet dew point, (c) CO production faradaic efficiency, (d) partial CO current density.

The imaging set-up is not suited for a controlled assessment of the electrochemical performance, including product gas analysis. Therefore, CO faradaic efficiency and electrochemical performance were separately evaluated in an analogue laboratory scale cell (4 cm^2^ active area). Fig. 6a shows the current density over time at constant voltage (3.4 V) for the different BPM variants. The current densities achieved are in the range where structural damage has been observed by XTM in the miniature cell (>50 mA cm^−2^). Most samples, except for the *commercial* variant, show some decay in current density over time during the potential hold, but no catastrophic failure during the observation period. The current densities (Fig. 6a) for the different membrane porosities show about an inverse trend with their faradaic efficiency (FE) to produce CO (Fig. 6c). Thus, the FE for the *custom*/*microporous*/*macroporous* AELs is below 20%. The *microporous* BPM-variant, combined with the serpentine flow field and to 5 bar CO_2_ pressure, achieves a high FE (about 80%) comparable to the *commercial* BPM, and even surpasses it in terms of CO partial current density (Fig. 6d). The only product other than CO is H_2_, as expected for the used Ag catalyst. Comparing the *microporous* layer with varying flow-field designs showed similar performance at low voltages, with differences only emerging at higher current densities (Fig. S34a and S34d) We also observe different water transport behaviour. In general, droplets could regularly be observed in the cathode outlet tubing, indicating liquid water being pushed out of the cell, with the cells with porous AEL showing qualitatively wetter outlet streams, as compared to the *custom* BPM. This observation is backed by the measured cathode outlet dew points, which indicate the presence of liquid water in the gas stream (Fig. 6b). All BPM variants, except for the *commercial* BPM, show fluctuating dew points, pointing to liquid water at the outlet and therefore increased water transport from anode to cathode. We finally observe a large extent of delamination outside the active area for the *custom* BPM after cell disassembly (Fig. S2).

## 4 Discussion

While recent developments^26,29,38–41^ in the design of FB-BPM CO_2_ reduction showed great improvements, our design still allows to discuss fundamental transport and degradation processes. In particular, results obtained here with the laboratory X-ray scanner and a Ti porous transport layer (PTL) at the anode reproduce well the observations on the same setup during our synchrotron campaign^23^ for the *commercial* BPM with a GDL at the anode. The systematic approach with variable current density and fixed passed charge allows us to identify a low but non-zero threshold current density at which BPM delamination occurs. The threshold current density, though, is material and cell design dependent. Furthermore, the developing ACL damage is independent of the applied current density and appears to correlate with the passed charge. Both the systematically reproduced current-induced BPM delamination and charge-induced ACL damage are strong indications to confirm our proposed model^23^ of CO_2_ transport pathways and gas-induced degradation: if the current-dependent CO_2_ production rate at the BPM interface is higher than the diffusive CO_2_ removal, accumulating CO_2_ forms gas pockets at sufficient pressure to separate the membrane layers. The CO_2_ pressure depends directly on the balance of CO_2_ production rate and back-diffusion, and builds up instantaneously with the set current density. Membrane delamination is therefore an immediate degradation process. A similar, but cumulative, process happens at the ACL-CEL interface. CO_2_ migrates to the anode and is trapped between the membrane surface and the water-filled nano-porous ACL. Pressure builds up until the ACL breaks, forming holes. Other than the membrane delamination, ACL damage is driven by slower accumulation of CO_2_ crossing the CEL by diffusion.

The fiber reinforcement of the *commercial* BPM-variant strengthens the membrane substantially against delamination compared to a simple *custom* bilayer composition. While the non-ion-conduction PEEK-fibers block part of the active area for direct ion migration, it is unclear how severely they impact performance. The manufacturer Fumasep provides negligible differences in specific ion conductivity ranges for AEM with and without fiber reinforcement (*e.g.* FAA-3-,-30,-50,-PK-75,-PK-130). Employing a Ti-PTL at the anode instead of a carbon GDL increases stiffness and the required pressure to delaminate the membrane. However, this slight increase in resistance against delamination is not enough to handle the relevant current densities beyond 200 mA cm^−2^.

To effectively avoid the BPM delamination, a CO_2_ escape route from the interface needs to be introduced by increasing the permeability of the AEL, either by employing a thinner or altogether porous AEL.^18,38^ However, recent studies^26,41^ show stable FB-BPM COELY operation also with a non-porous AEL. To put these works into context, we employ our previously developed model^23^ to estimate the pressure at the AEL-CEL interface of the BPM for different AEL thicknesses and operating conditions including differential pressure (SI). Similar to reinforcing the BPM, we find combinations of materials and operating conditions that could potentially prevent BPM delamination by increased CO_2_ back-diffusion and pressurizing the cathode countering the CO_2_ pressure build-up within the membrane. Especially at current densities above 1 A cm^−2^, the balance between the different mechanisms is delicate, sensitive to material properties and requires careful consideration to prevent damage to the BPM. A porous AEL, on the other hand, can ensure efficient CO_2_ back-diffusion with less effect on other membrane properties like thickness and ion mobility. All BPM-variants with a porous AEL prevent delamination within our tested conditions. While effective, the *macroporous* and *mesoporous* variants are limited in their applicability. The loss of active area in the *macroporous* case by punching almost mm-sized holes into the AEL already corresponds in magnitude to the damage in the *commercial* case. The CNC-process in the *mesoporous* case gives a very controlled pinch pattern but is infeasible for anything larger than the tested 2 mm × 5 mm active area. The pattern and introduced AEL puncture itself is similar to the method proposed by Disch *et al.*^18^ with a needled roller, which might be developed into a controlled technical solution.

The *microporous* variant shows the best resistance to gas-induced degradation, as we do not only observe any BPM delamination but also much lower ACL damage compared to all other cases. As already outlined in the methods and results sections, there is a non-negligible uncertainty to ACL-damage quantification (Fig. S33).

For the *macroporous* and *mesoporous* variants, the AEL does not offer open gas percolation except for the introduced holes. CO_2_ produced at the interface has first to diffuse in-plane to reach an opening in the AEL. The consequences are local gradients of CO_2_ concentration similar to the non-porous variants. The CO_2_ concentration gradients promote CO_2_ cross-over to the anode and accumulation of CO_2_ at the CEL-ACL interface, causing ACL holes. Since the *mesoporous* membrane is not delaminating, the CO_2_ concentration gradient from the AEL-CEL interface to the anode might by stronger than in the dense *custom* and *commercial* cases, but this is not entirely clear without further investigation. The advantage of the *microporous* BPM lies in the availability of percolation pathways back to the cathode at each point of the BPM interface.

For a more thorough analysis, we suggest a dedicated XTM imaging campaign at a synchrotron source tailored to tackle the limitations of tube-source XTM, *i.e.* higher spatial resolution, better statistics through more data points per sample with time-resolved experiments, optimized X-ray spectrum, and parallel beam geometry to reduce metal artifacts.

Low FE for CO and a vapor-saturated gas outlet with liquid droplets are strong indications for a water-flooded cathode shifting from CO_2_ reduction to hydrogen evolution. However, since the FE is not zero for all samples, we can deduce that the cathode is only partially flooded. The cathode, therefore, also allows the formation of (bi)carbonate ions as charge carriers in the AEL and the formation of CO_2_ at the BPM interface in all cases (Fig. 1). We additionally do not expect any depletion of CO_2_ at the cathode because inlet CO_2_ flux is abundant compared to the achieved current densities: The CO_2_ stoichiometry factor is above 8 assuming a current density of 100 mA cm^−2^ and FE = 100% for the laboratory scale cell measurements.

The *microporous* BPM not only prevents CO_2_ gas-induced damage, but also shows promising electrochemical performance among the tested variants in the laboratory scale fixture. The initially rather low FE of the *microporous* BPM can be improved to the same level as the *commercial* BPM by using a serpentine flow field and increasing the pressure in the cell. Introducing a porous AEL and using Nafion as CEL greatly changes the water permeability of the BPM and, consequently, the water management of the full cell.^42^ Forcing part of the gas stream through the GDL below the ribs in the serpentine flow field can push liquid water out and facilitate the access of CO_2_ to the catalyst layer. The additional positive effect of increasing the CO_2_ pressure from 1 bar to 5 bar may be explained by the change of the wetting behavior in the CL. The capillary pressure is the difference between the liquid and gas phases. An *ex situ* capillary pressure of 4 bar can, by approximation with the Young–Laplace equation, move the water–gas interface into water-wet pores bigger than 700 nm, which covers a larger part of the CL pore space^45^ than at negligible capillary pressure, effectively allowing greater access to catalytic sites. The actual *in situ* pressure difference between the liquid and gas phases, as well as potential transient variations, remains to be investigated. Enthalpy related to the transition from molecular water (vapor, bound in the ionomer matrix) to liquid, reaction enthalpies including the increased partial CO_2_ pressure, surface wetting in the complex CL geometry, and dynamic gas pressure will affect the free energy balance and therefore pressure difference.^46–48^

Overall, the performance of all tested BPM variants still stays below commercially relevant power densities in the 1 A cm^−2^ range at low (<3 V) voltages. A porous AEL appears to be a highly desirable, but not sufficient modification for the success of forward bias BPM CO_2_ co-electrolysis.

## 5 Conclusion

We employed X-ray tomographic microscopy (XTM) to systematically study the origin and effect of gas-induced structural degradation in forward bias (FB) bipolar membrane (BPM) CO_2_ electrolysis. We tested different porous anion exchange layers in the BPM as a modification to prevent membrane delamination and anode catalyst layer damage by enhancing CO_2_ transport back to the cathode.

Our quantitative study reproduces previous qualitative results and confirms the previously postulated model of CO_2_ pathways in FB-BPM-CO2ELY. BPM delamination occurs if the CO_2_ production by ion recombination at the BPM interface is greater than the diffusive removal through the BPM layers to the cathode (back diffusion) and anode (crossover). Different porous anion exchange membranes for the BPM have been evaluated for enhanced back transport of CO_2_ to the cathode. The ideal porous AEL has a high density of percolation paths from the BPM interface to the cathode to avoid in-plane transport and build-up of CO_2_ concentration at the BPM membrane interface. By spraying an ionomer-nanoparticle mixture, a fully porous AEL was prepared, other than the previous locally perforated, but otherwise dense AEL.^29,38^

Our study shows that preventing gaseous CO_2_ formation within the BPM is a crucial aspect for the long-term stable operation. The preparation of a *microporous* layer has a manufacturing approach that is scalable and compatible with catalyst layer-like processing techniques. Further optimization of *microporous* AEL will include ink composition optimization, *e.g.* including hydrophobic nanoparticles to prevent flooding.

## Author contributions

Robert Fischer: conceptualization, methodology, formal analysis, investigation, writing – original draft, visualization. Matthieu A. Dessiex: conceptualization, methodology, formal analysis, investigation, writing – original draft, visualization. Lorenz Gubler: conceptualization, writing – review & editing. Sophia Haussener: conceptualization, writing – review & editing, supervision, project administration, funding acquisition. Felix N. Büchi: conceptualization, methodology, writing – review & editing, supervision, project administration, funding acquisition.

## Conflicts of interest

There are no conflicts to declare.

## Supplementary Material

TA-013-D5TA04879F-s001

## Data Availability

The experimental data (imaging and electrochemical) is deposited to the Paul Scherrer Institut public data repository (https://doi.psi.ch/) under the following doi: https://doi.org/10.16907/b1d794ed-d89c-4e09-a7f3-77ab5200fad7 Supplementary information: Supporting X-ray images and data plots referenced in the main text. See DOI: https://doi.org/10.1039/d5ta04879f.
