# Supplementary material for: Mitigation of gas-induced damage in bipolar membranes for CO2 electrolysis
Source: J Mater Chem A Mater. 2025 Sep 12;13(39):33924–34. doi: 10.1039/d5ta04879f (PMC12426753; doi:10.1039/d5ta04879f)
Supplement: TA-013-D5TA04879F-s001 [file TA-013-D5TA04879F-s001.pdf]

Supplementary Information (SI) for Journal of Materials Chemistry A.
This journal is © The Royal Society of Chemistry 2025

Supporting Information to: Mitigation of gas-induced damages in bipolar membranes for CO_2 Electrolysis

Robert Fischer[‡,1], Matthieu A. Dessiex[‡,1,2], Lorenz Gubler[1], Sophia Haussener[2], and Felix N. Büchi[1]

[‡]contributed equally
[1]PSI Center for Energy and Environmental Sciences, 5232 Villigen PSI, Switzerland
[2]Laboratory of Renewable Energy Science and Engineering, Ecole Polytechnique Fédérale de Lausanne (EPFL), 1015 Lausanne, Switzerland

September 1, 2025

1 Microscopy images of modified membranes

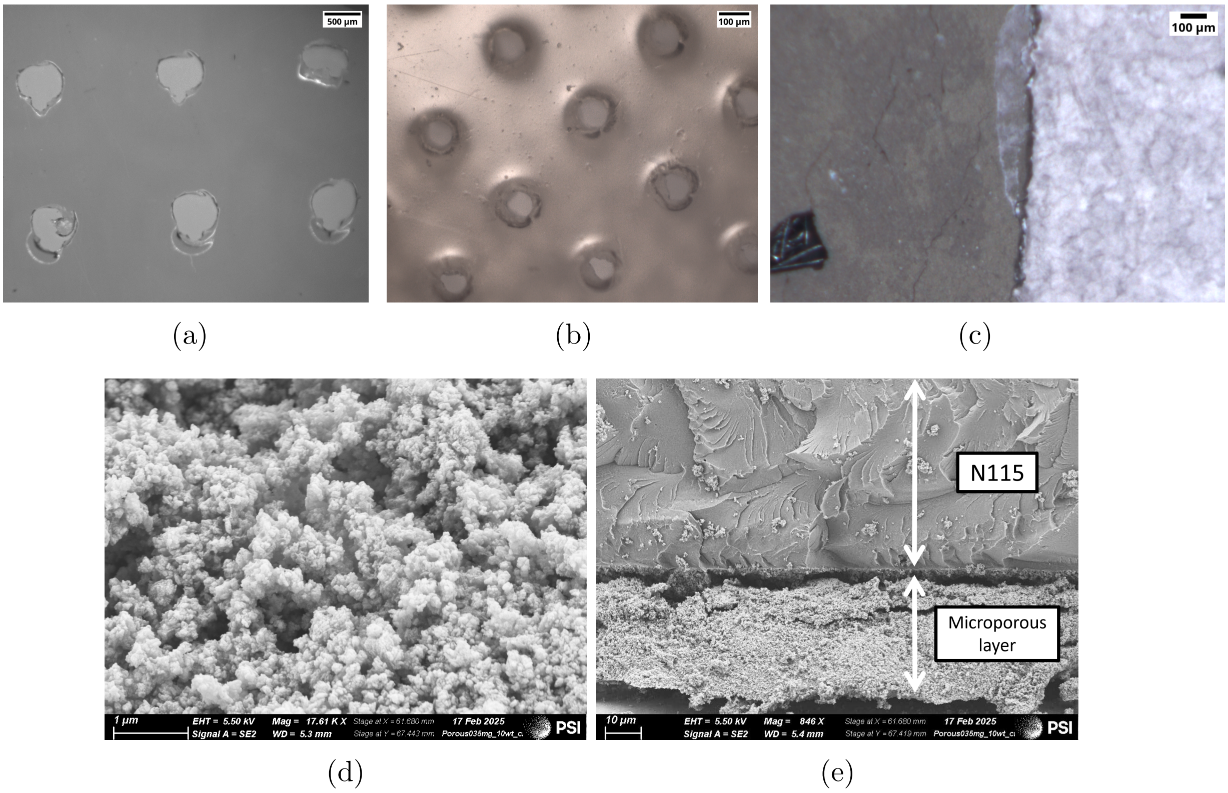

Figure S1: Microscopic images of the employed porous AEL. a) *macroporous*, b) *mesoporous*, c) the white layer on the right is the *microporous* layer sprayed directly on the cathode CL (brownish-gray layer on the left). d-e) cross-section SEM of the *microporous* layer

2 Anode catalyst layer trace extraction

1. all images were normalized by grayscale shifting. The shift is the difference of the median of the through-plane maximal grayvalues from a common reference, i.e. to achieve a common CL brightness over all images
2. a raw ACL segmentation was obtained by 2D (orientation normal to the channels) machine learning (ML) segmentation (Weka in ImageJ) [1]
3. the raw CL segmentation is expanded to a thick search band by
   (a) 3D binary opening (radius 1px) to remove spurious pixels
   (b) 3D binary closing (radius 35px) to close holes in the segmentation and create a thick search band
4. look for the maximum through-plane grayvalue maximum in the search band
5. expand the obtained binary spine to 3px with a binary median filter
6. fill vertical gaps to achieve a continuous 3px wide trace

3 Visually perceptual delamination

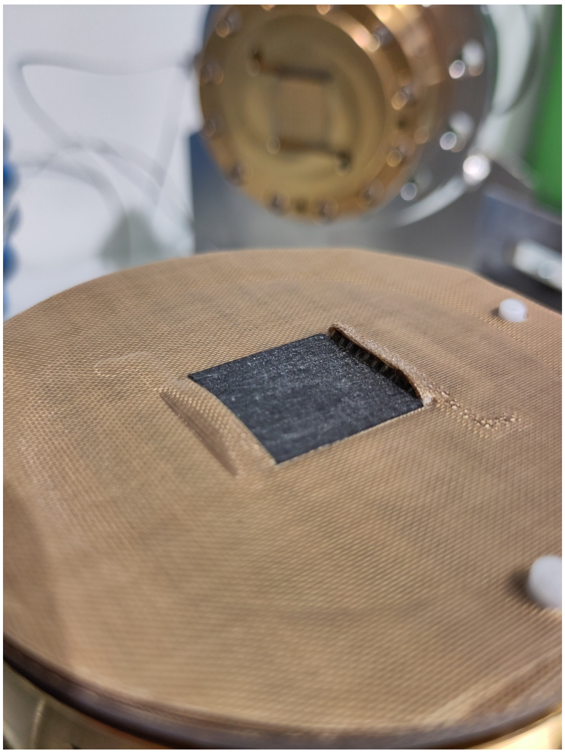

Figure S2: Photography of disassembled laboratory scale electrolyzer showing big inflated parts of the *custom* BPM at the edges of the active area after operation. The bloated membrane (transparent) is visible below the lifted Fiberflon (brown) gasket.

4 Isodistance images of all investigated samples

The following figures display the isodistance images that informed table 2 . The order corresponds to table 2, i.e. material type and current density in ascending order.

4.1 *Commercial* BPM with GDL at anode

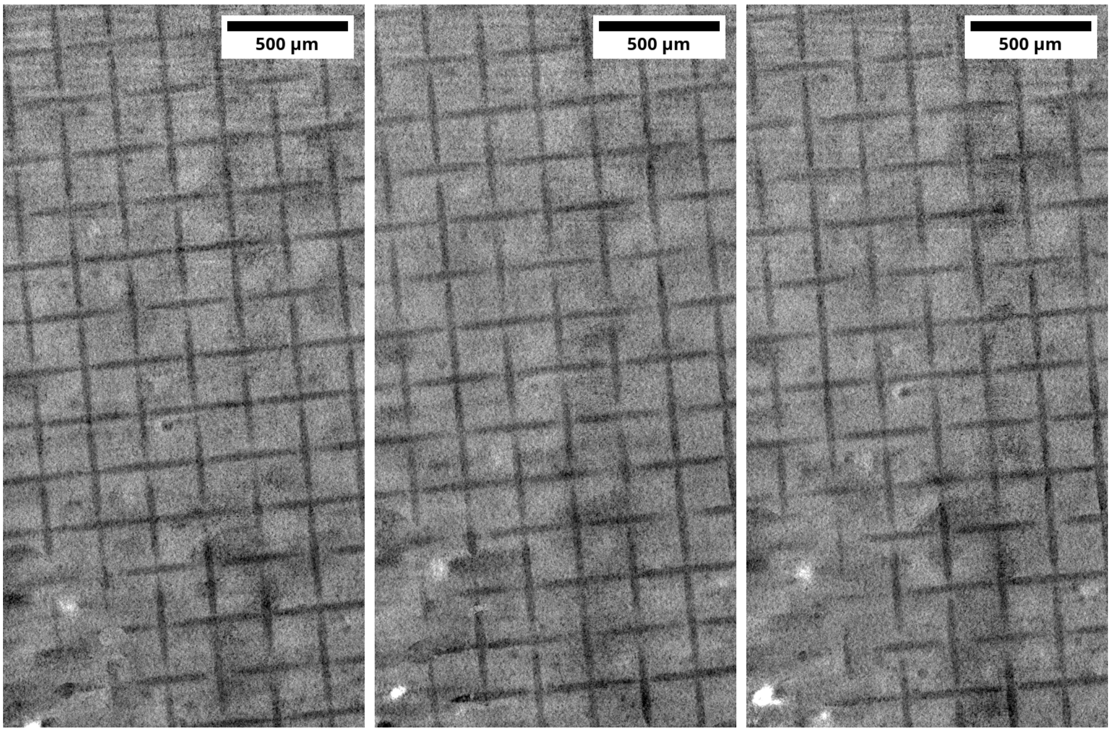

(a) BOL (b) 1st scan (c) EOL

Figure S3: 25mA/cm^2; *commercial* BPM with GDL at anode

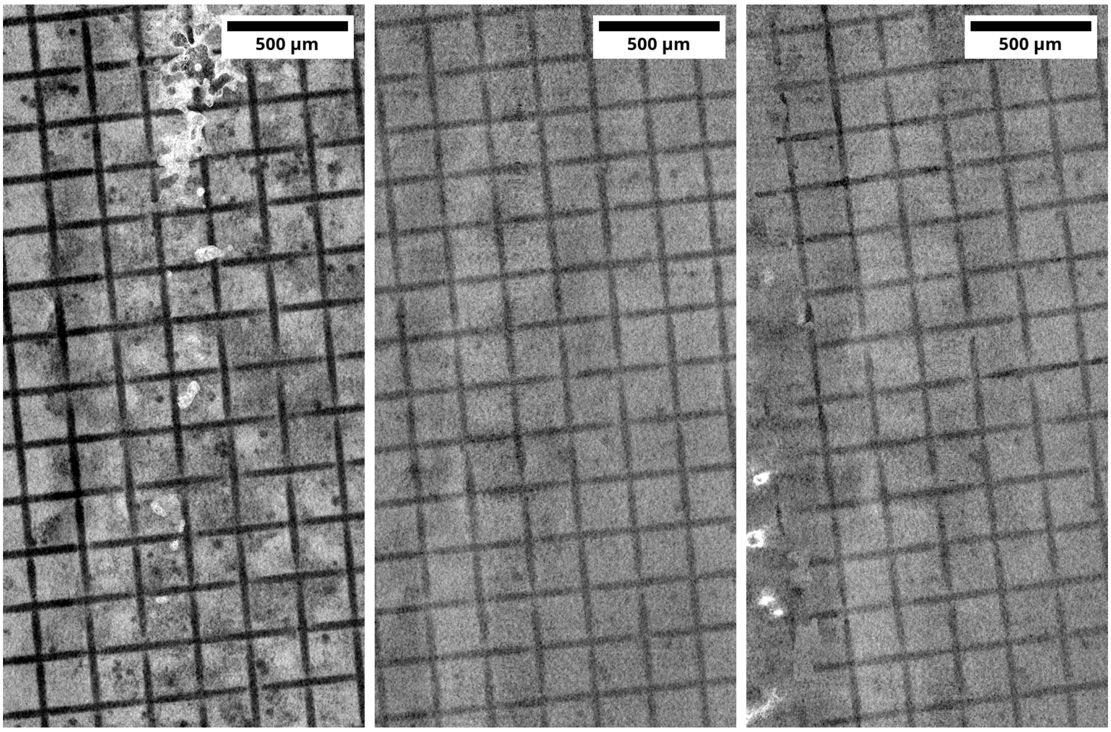

(a) BOL (b) 1st scan (c) EOL

Figure S4: 25mA/cm^2 repeat; *commercial* BPM with GDL at anode

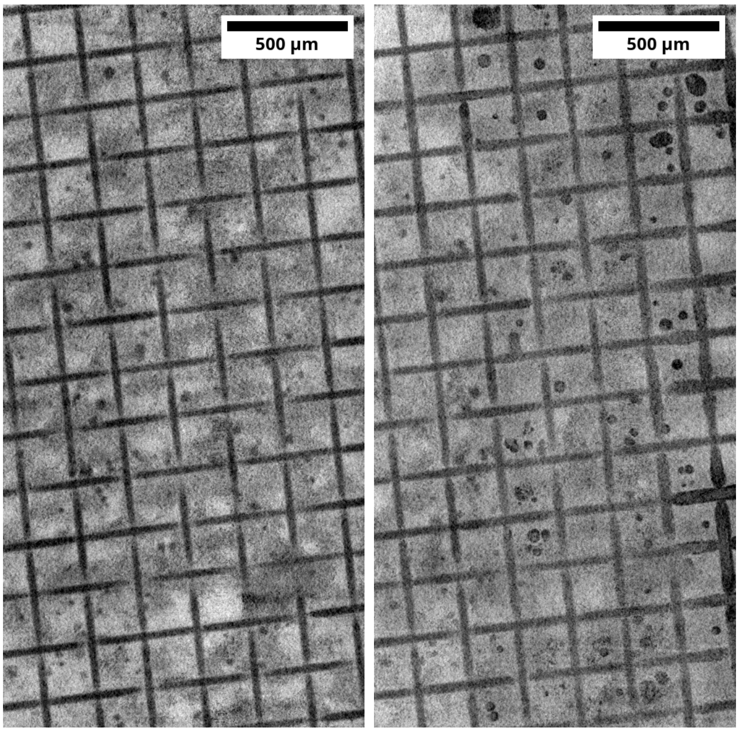

(a) BOL (b) 1st scan/EOL

Figure S5: max. 50mA/cm^2 (non-galvanostatic); *commercial* BPM with GDL at anode

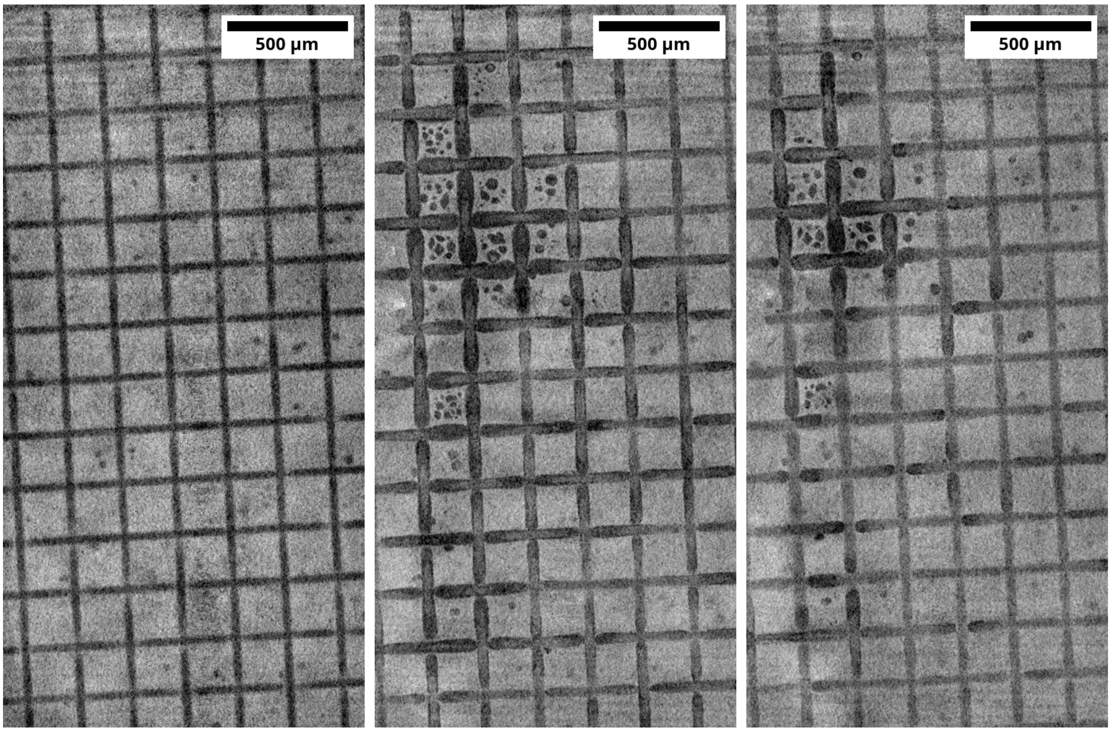

(a) BOL (b) 1st scan (c) EOL

Figure S6: 50mA/cm^2; *commercial* BPM with GDL at anode

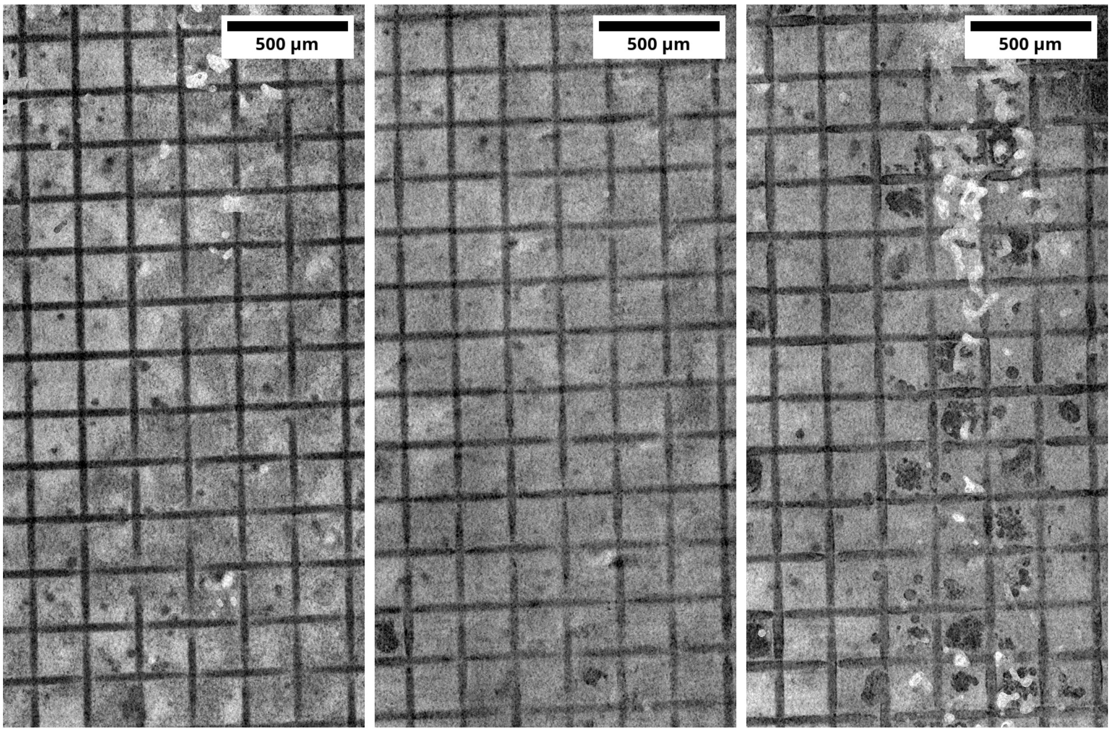

(a) BOL (b) 1st scan (c) EOL

Figure S7: 50mA/cm^2 repeat; *commercial* BPM with GDL at anode

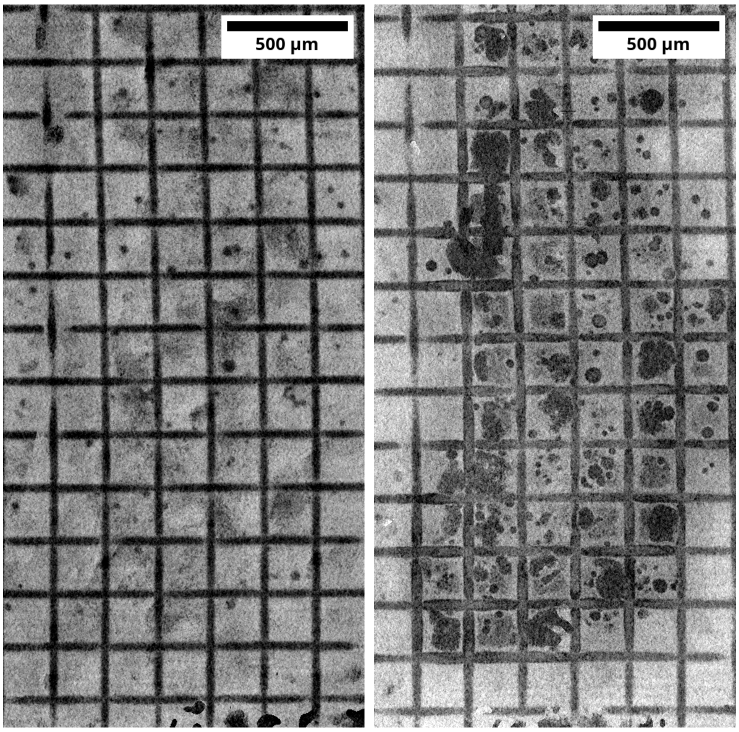

(a) BOL (b) 1st scan/EOL

Figure S8: max. 80mA/cm^2 (non-galvanostatic); *commercial* BPM with GDL at anode

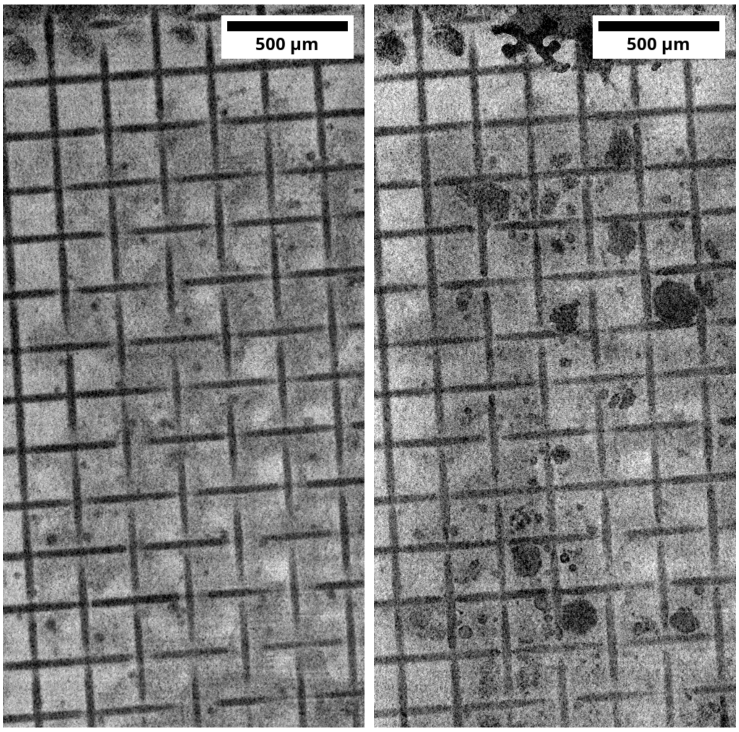

(a) BOL (b) 1st scan/EOL

Figure S9: max. 80mA/cm^2 (non-galvanostatic) repeat; *commercial* BPM with GDL at anode

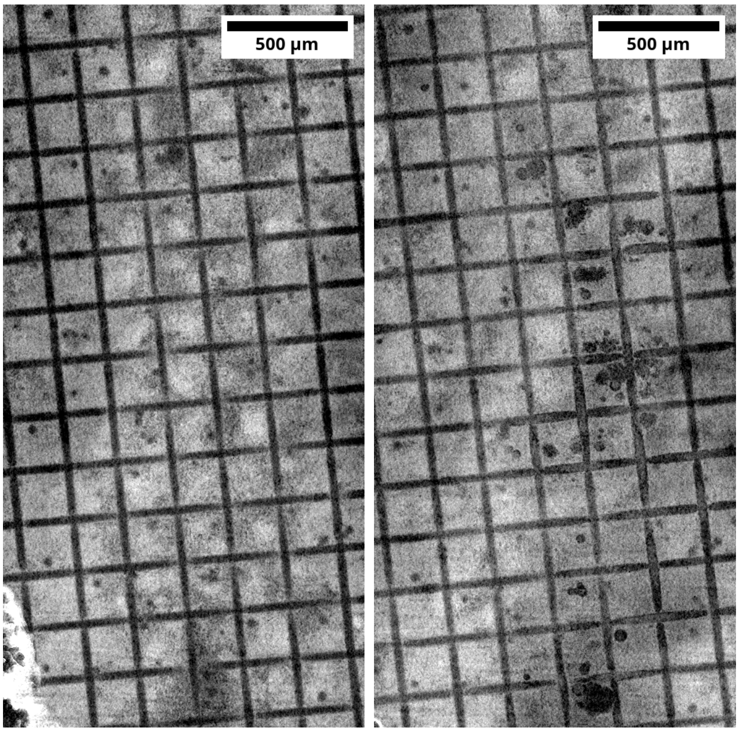

(a) BOL (b) 1st scan/EOL

Figure S10: max. 80mA/cm^2 (non-galvanostatic) 2nd repeat; *commercial* BPM with GDL at anode

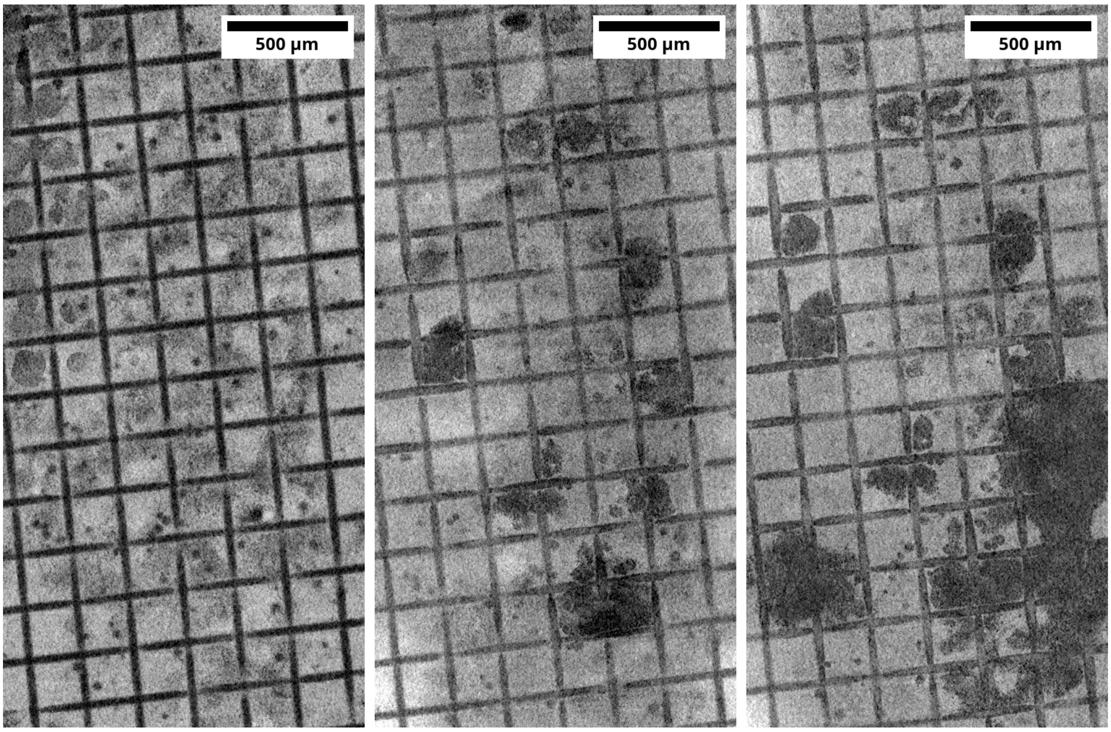

(a) BOL (b) 1st scan (c) EOL

Figure S11: 100mA/cm^2; *commercial* BPM with GDL at anode

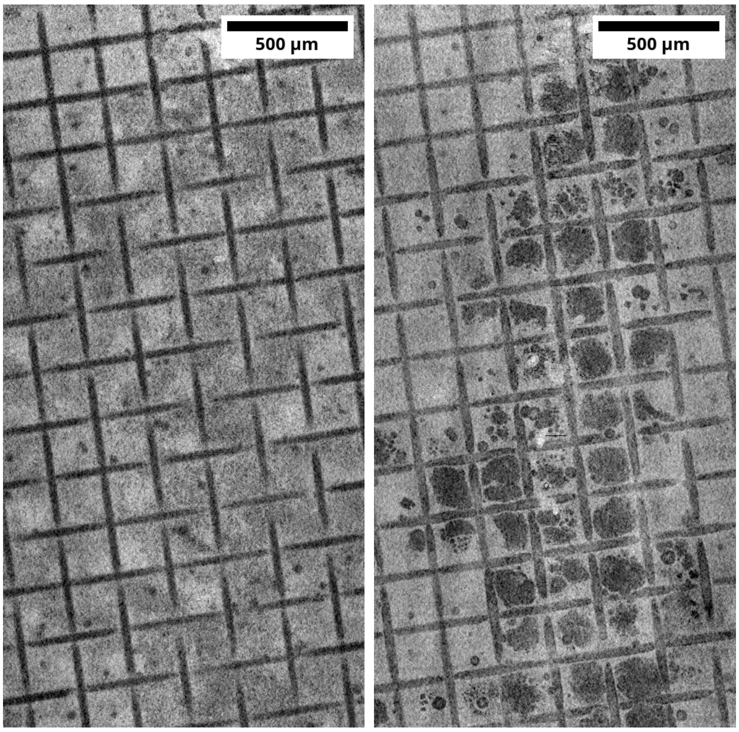

(a) BOL (b) 1st scan/EOL

Figure S12: >200mA/cm^2; *commercial* BPM with GDL at anode

4.2 *Commercial* BPM with Ti-PTL at anode

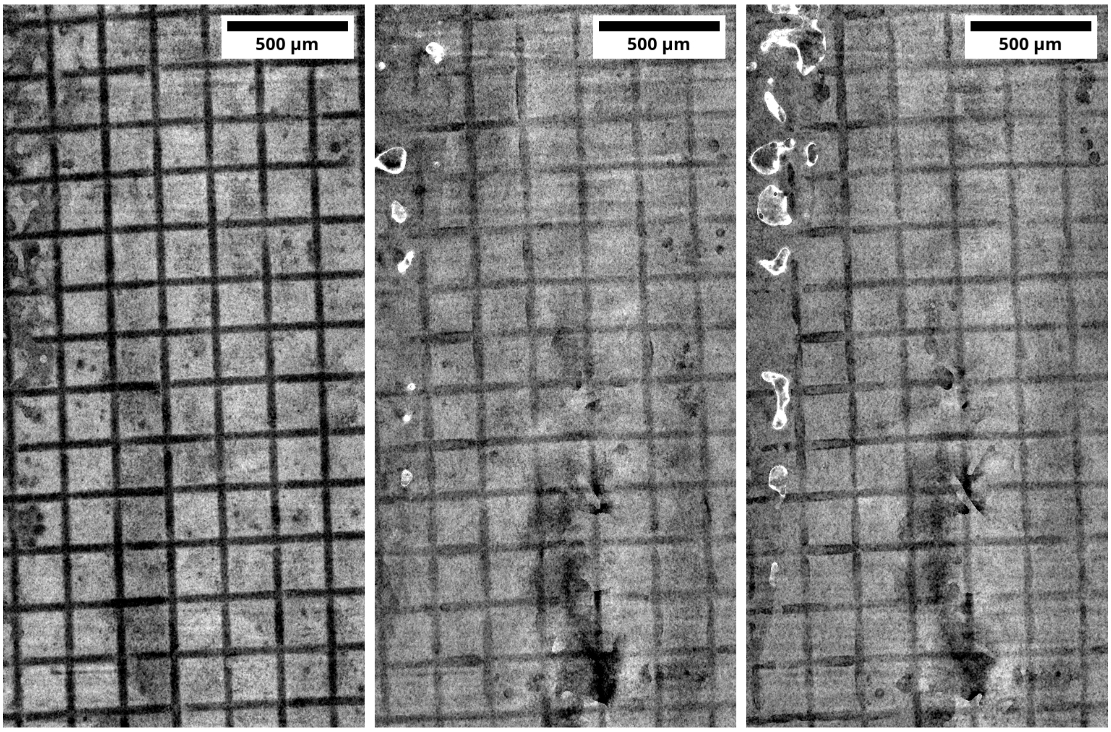

(a) BOL (b) 1st scan (c) EOL

Figure S13: 25mA/cm^2; *commercial* BPM with Ti-PTL at anode

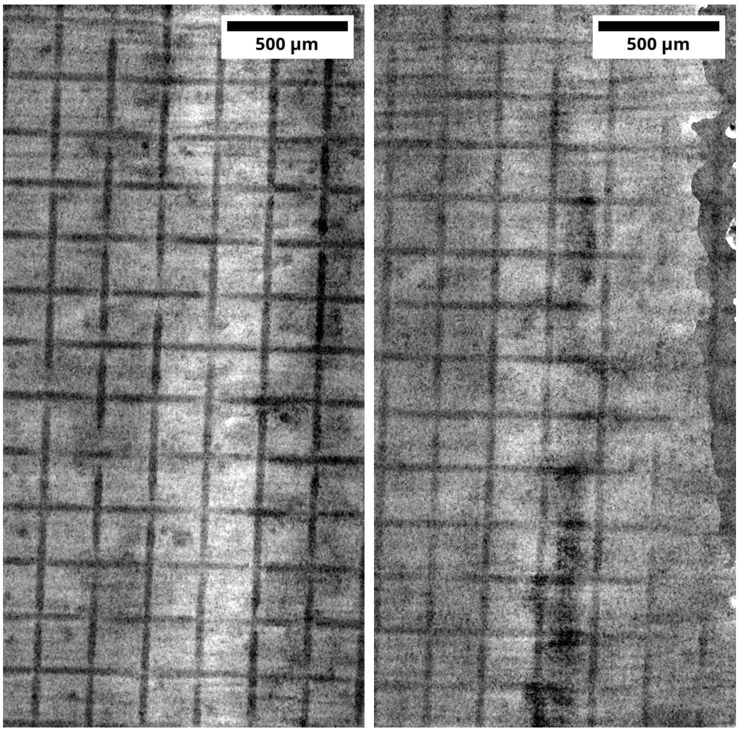

(a) BOL (b) 1st scan/EOL

Figure S14: max 50mA/cm^2 (non-galvanostatic); *commercial* BPM with Ti-PTL at anode

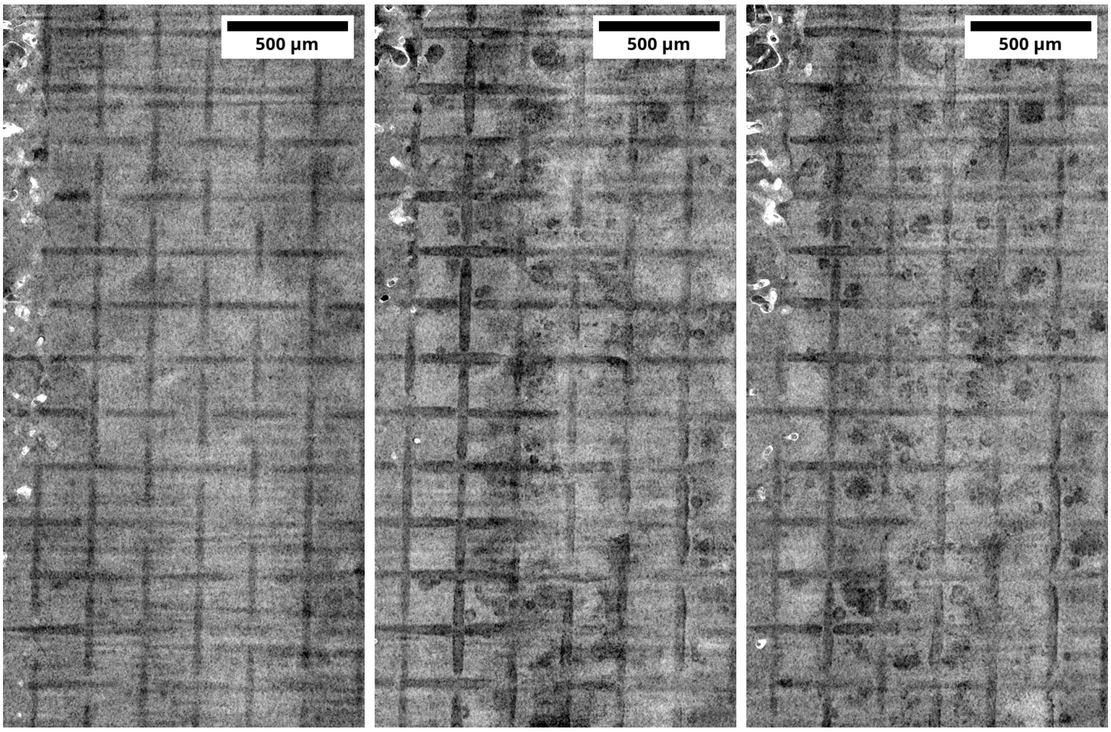

(a) BOL (b) 1st scan (c) EOL

Figure S15: 100mA/cm^2; *commercial* BPM with Ti-PTL at anode

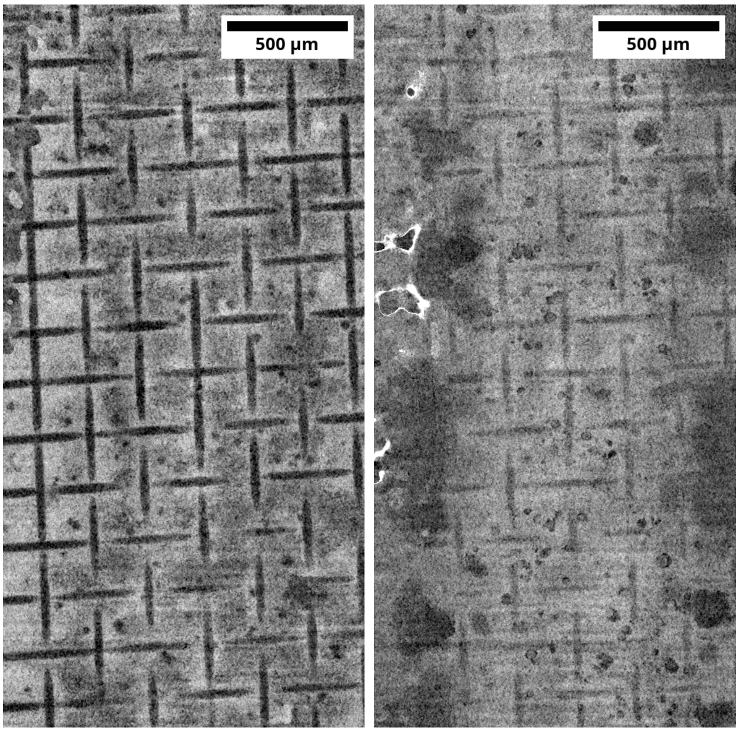

(a) BOL (b) 1st scan/EOL

Figure S16: 100mA/cm^2 repeat; *commercial* BPM with Ti-PTL at anode

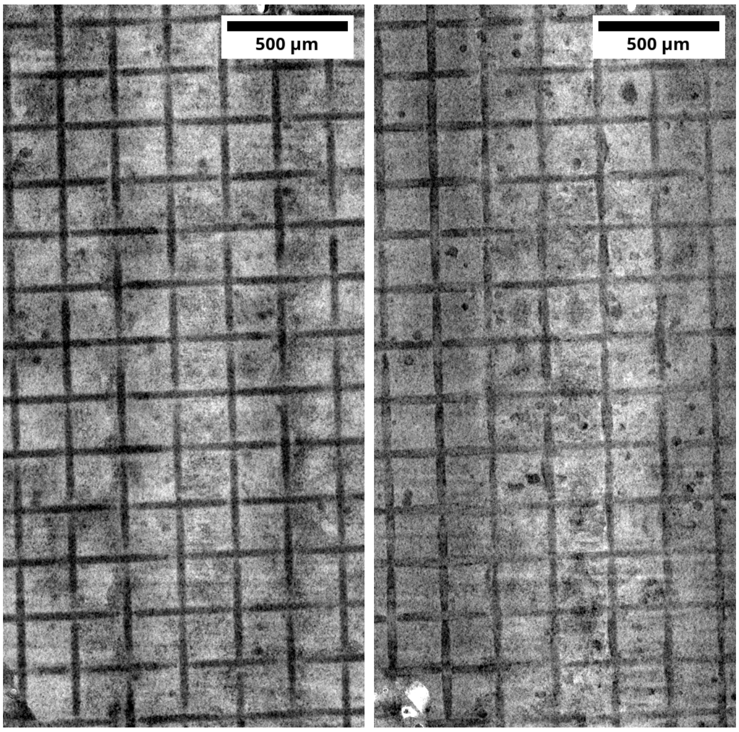

(a) BOL (b) 1st scan/EOL

Figure S17: 150mA/cm^2 repeat; *commercial* BPM with Ti-PTL at anode

4.3 *Custom*

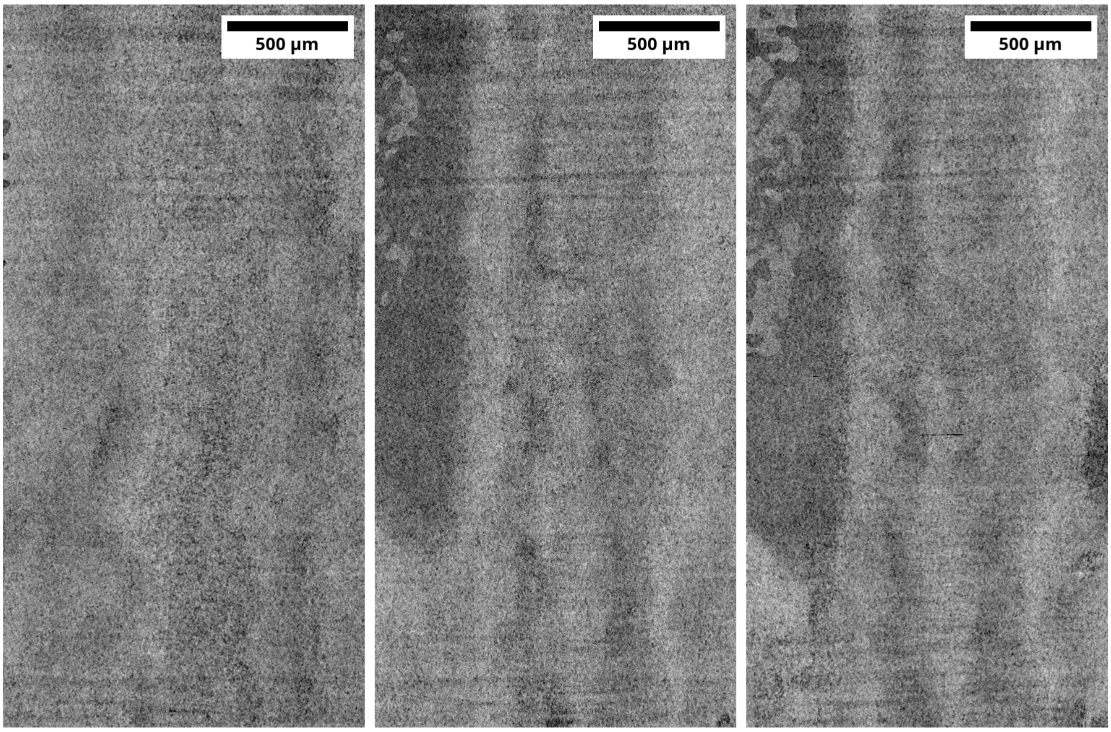

(a) BOL (b) 1st scan (c) EOL

Figure S18: 50mA/cm^2; *custom*

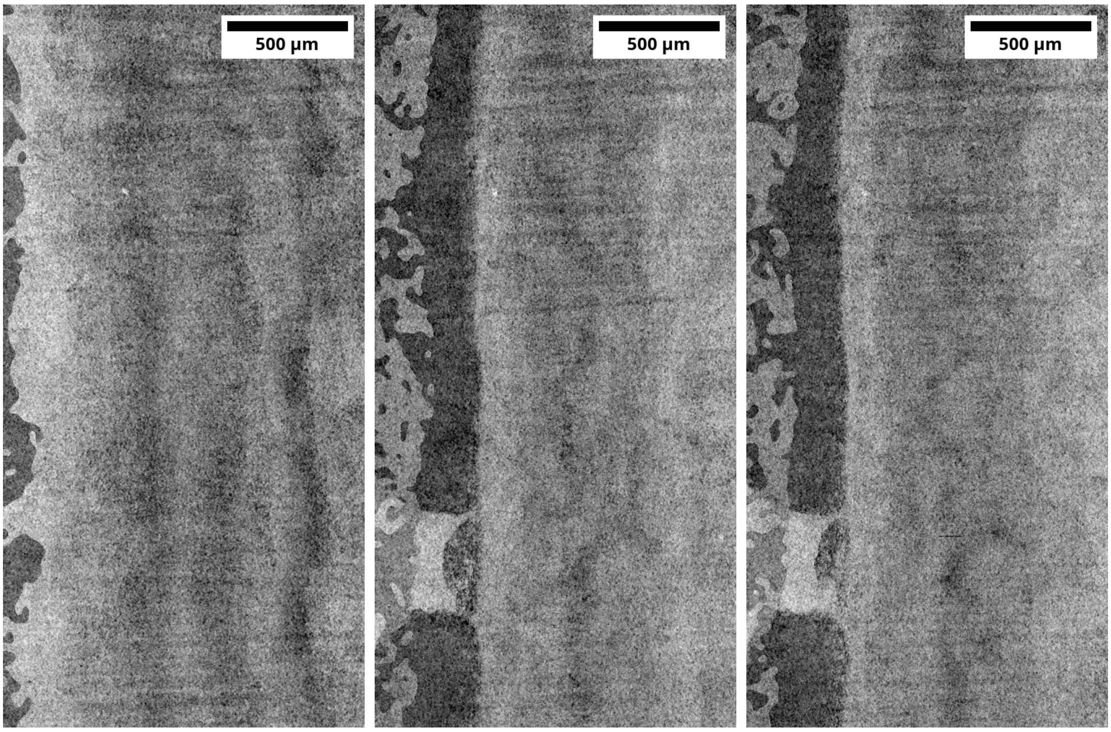

(a) BOL (b) 1st scan (c) EOL

Figure S19: 100mA/cm^2; *custom*

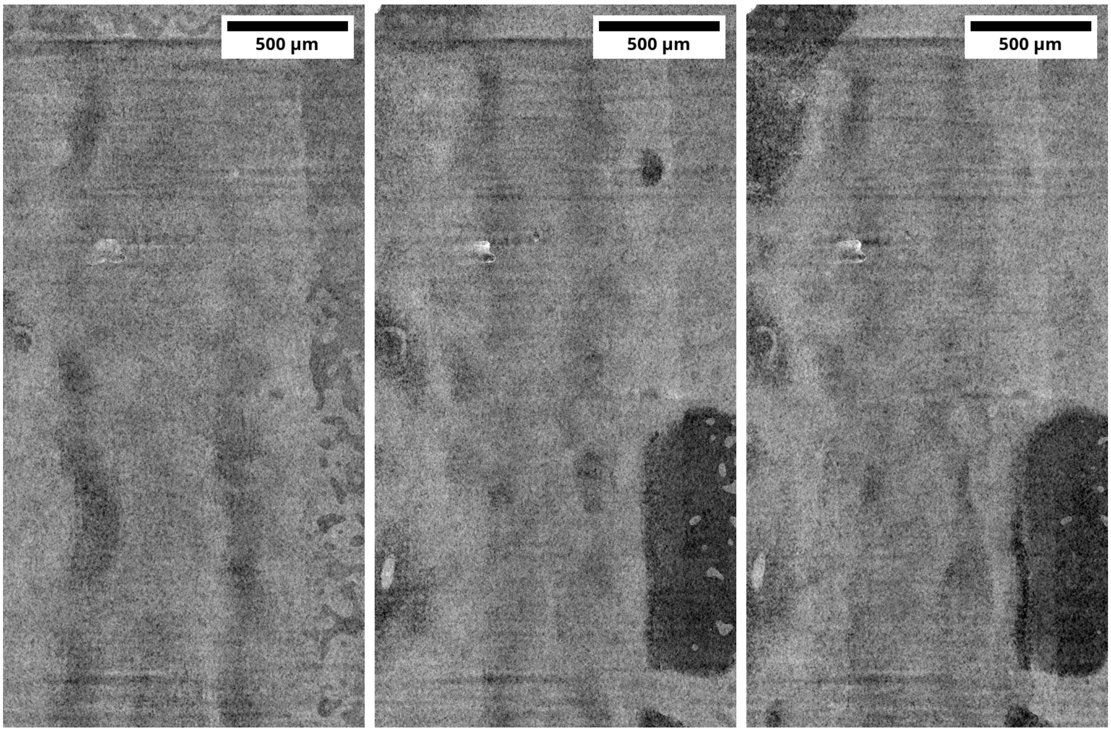

(a) BOL (b) 1st scan (c) EOL

Figure S20: >200mA/cm^2; *custom*

4.4 *Macroporous*

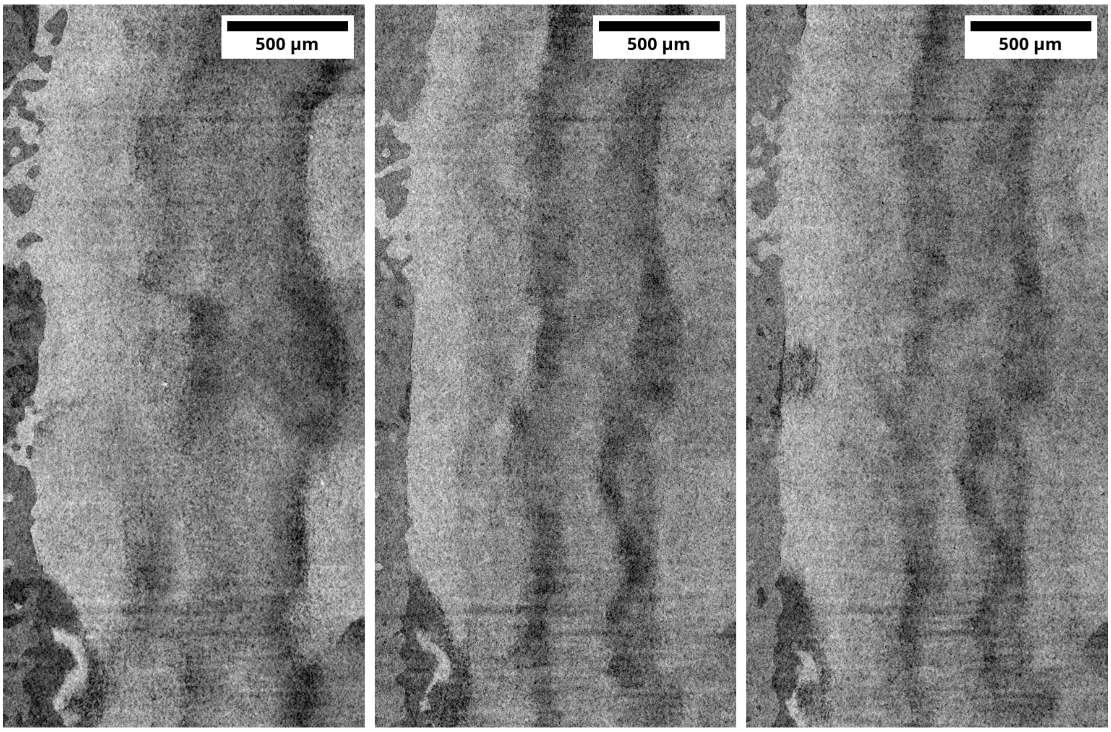

(a) BOL (b) 1st scan (c) EOL

Figure S21: 50mA/cm^2; *macroporous*

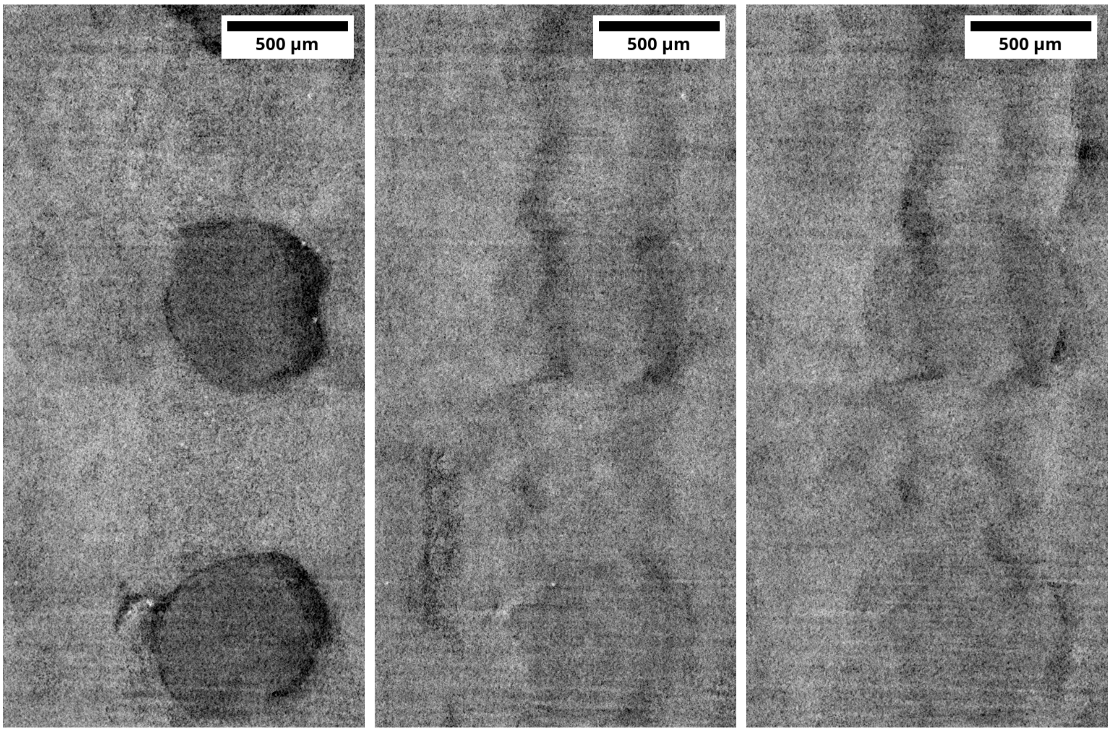

(a) BOL (b) 1st scan (c) EOL

Figure S22: 100mA/cm^2; *macroporous*

4.5 *Mesoporous*

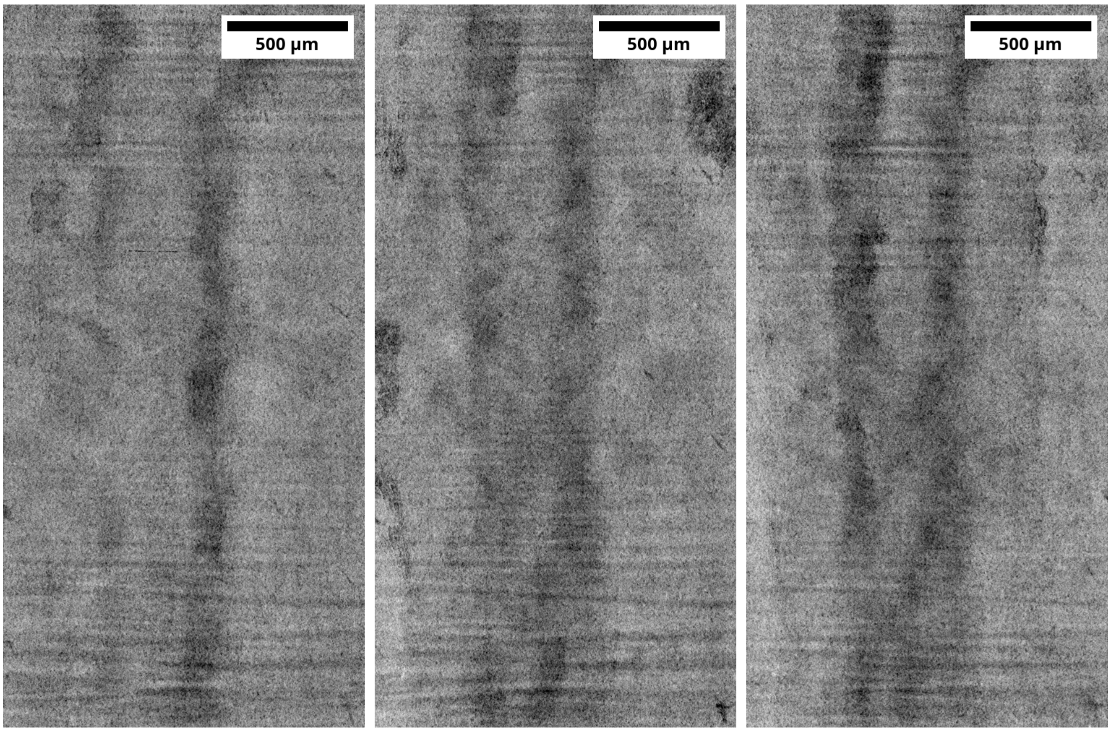

(a) BOL (b) 1st scan (c) EOL

Figure S23: max 80mA/cm^2 (non-galvanostatic); *mesoporous*

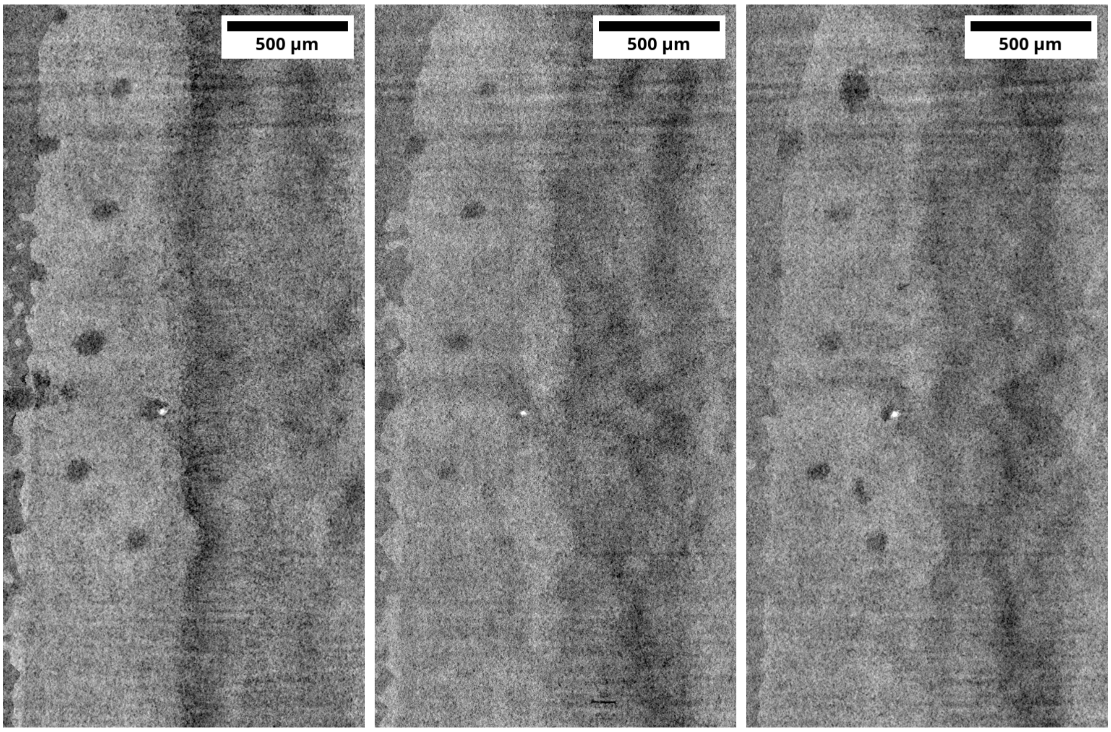

(a) BOL (b) 1st scan (c) EOL

Figure S24: 100mA/cm^2 ; *mesoporous*

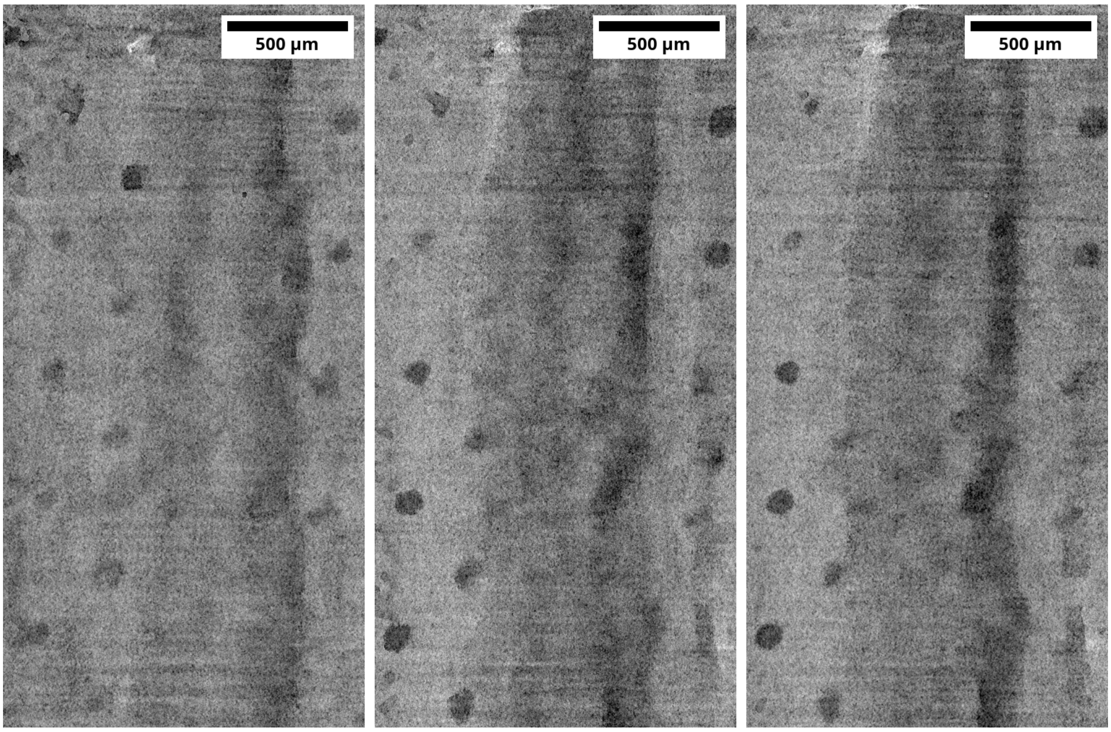

(a) BOL (b) 1st scan (c) EOL

Figure S25: 100mA/cm^2 repeat; *mesoporous*

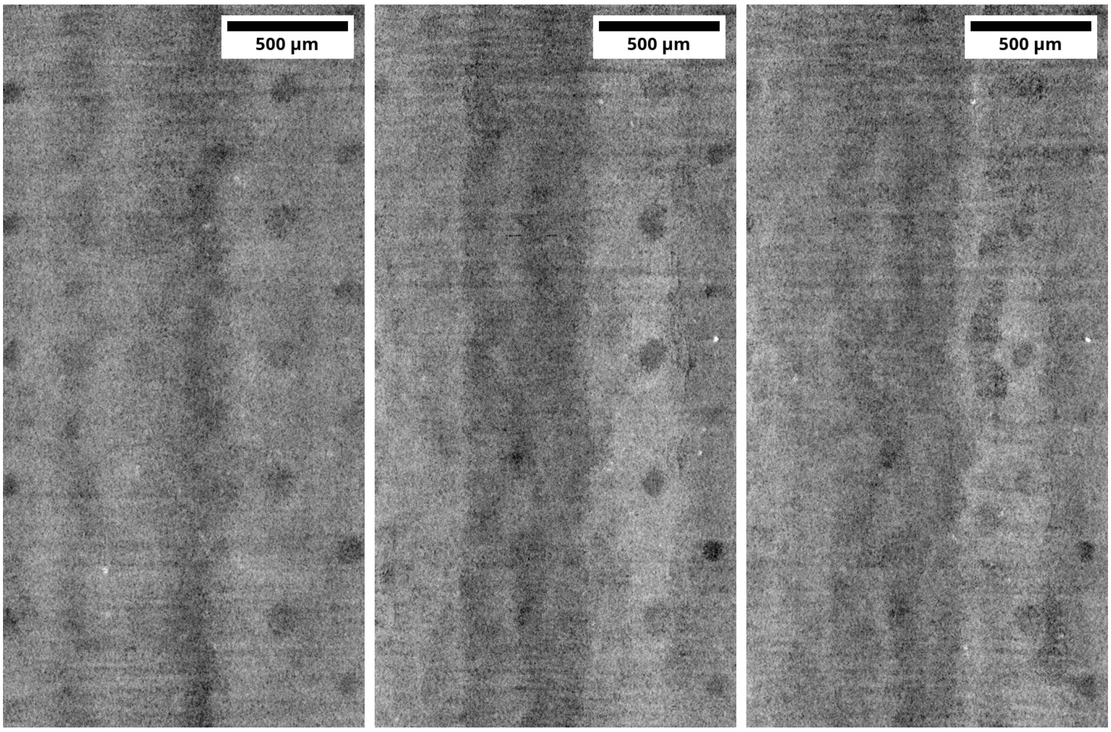

(a) BOL (b) 1st scan (c) EOL

Figure S26: >200mA/cm^2 ; *mesoporous*

4.6 *Microporous*

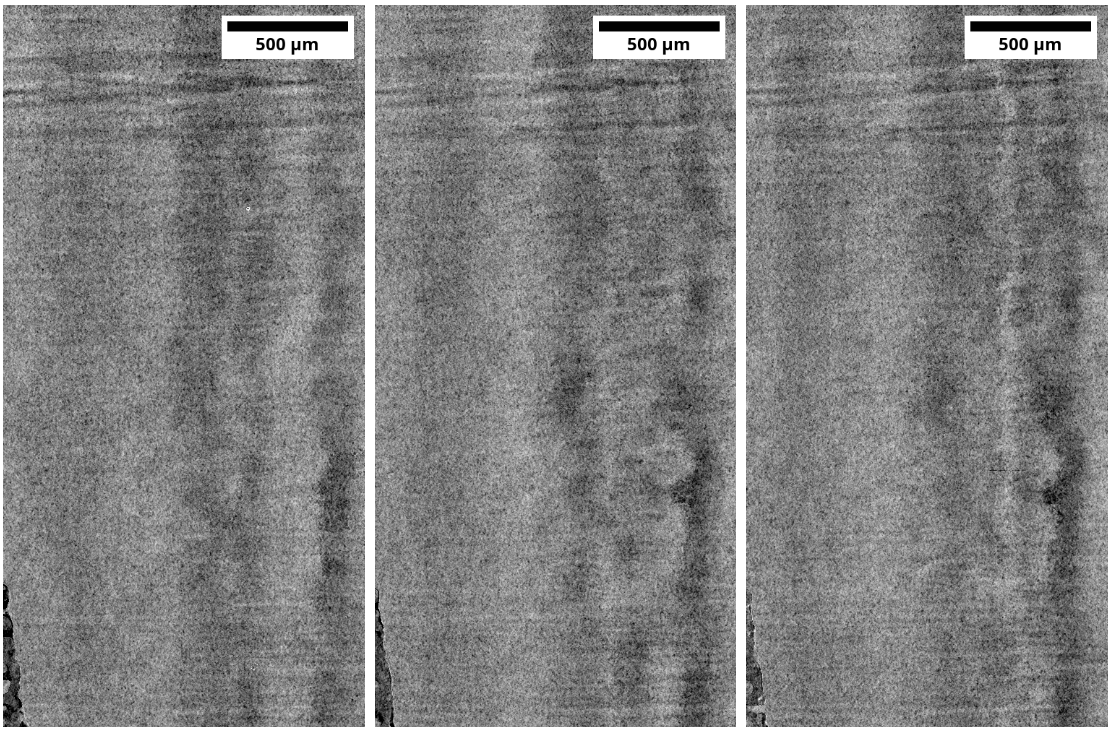

(a) BOL (b) 1st scan (c) EOL

Figure S27: 50mA/cm^2; *microporous*

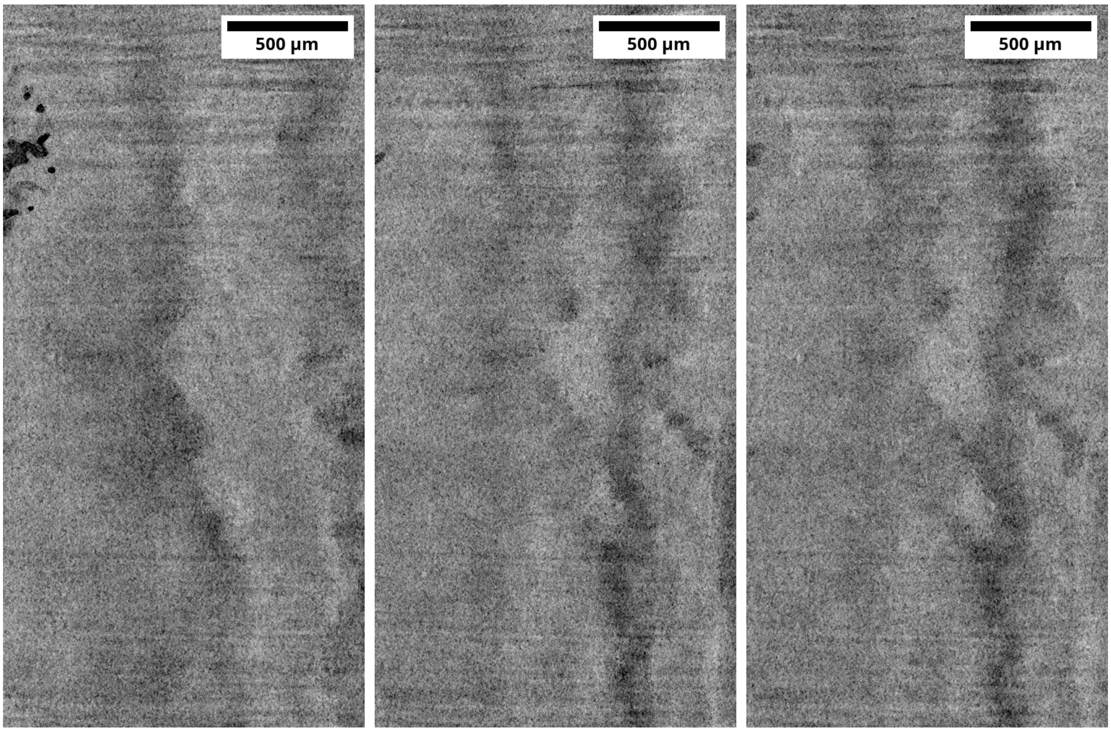

(a) BOL (b) 1st scan (c) EOL

Figure S28: 50mA/cm^2 repeat; *microporous*

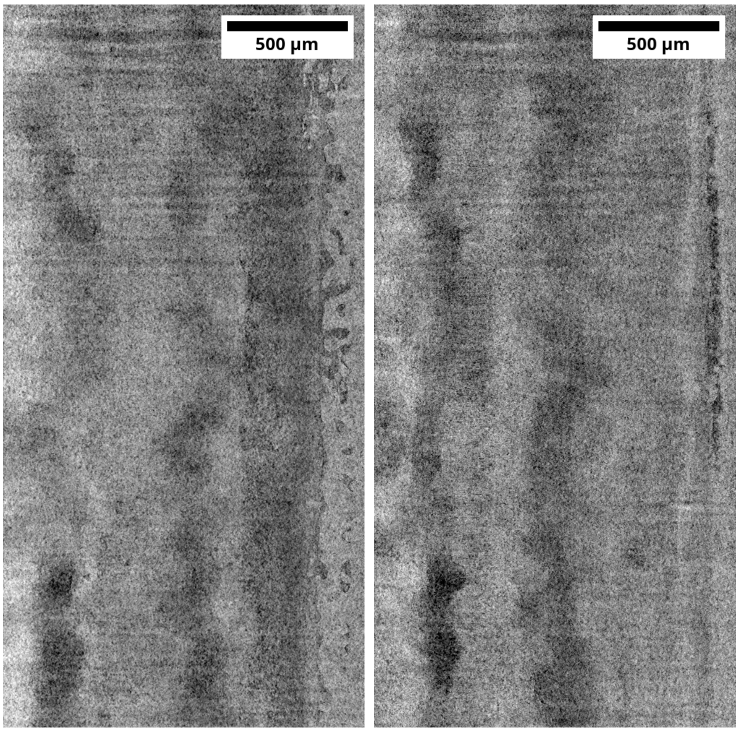

(a) BOL (b) 1st scan/EOL

Figure S29: 100mA/cm^2; *microporous*

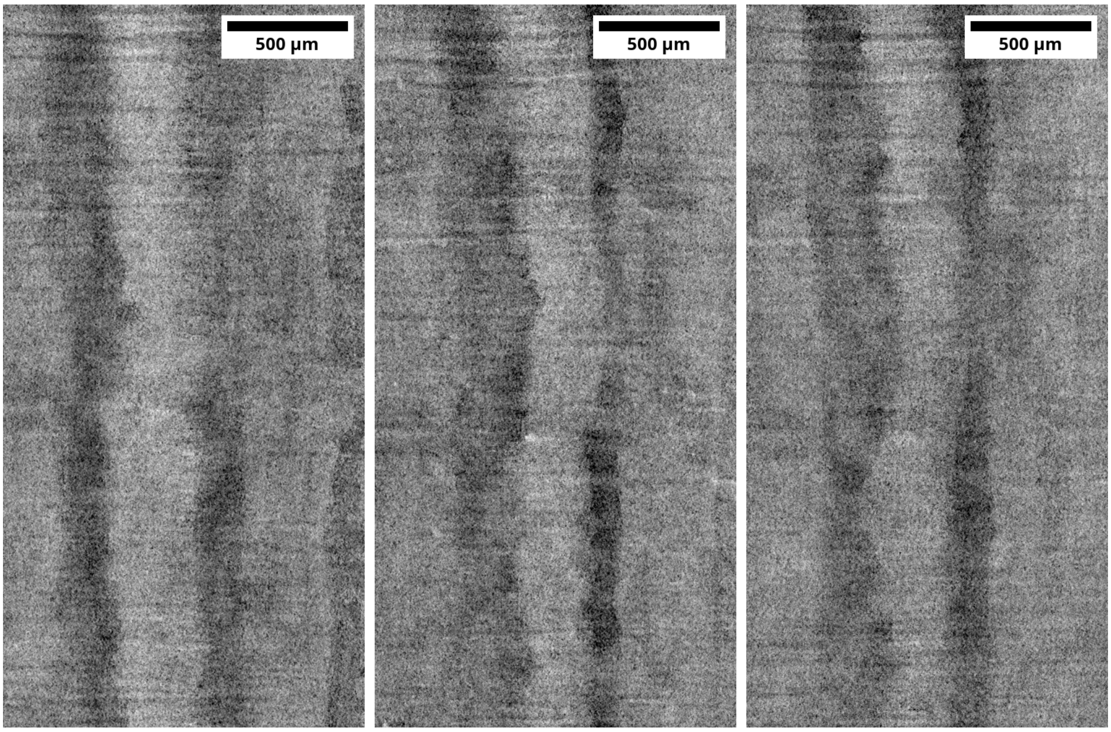

(a) BOL (b) 1st scan (c) EOL

Figure S30: 100mA/cm^2 repeat; *microporous*

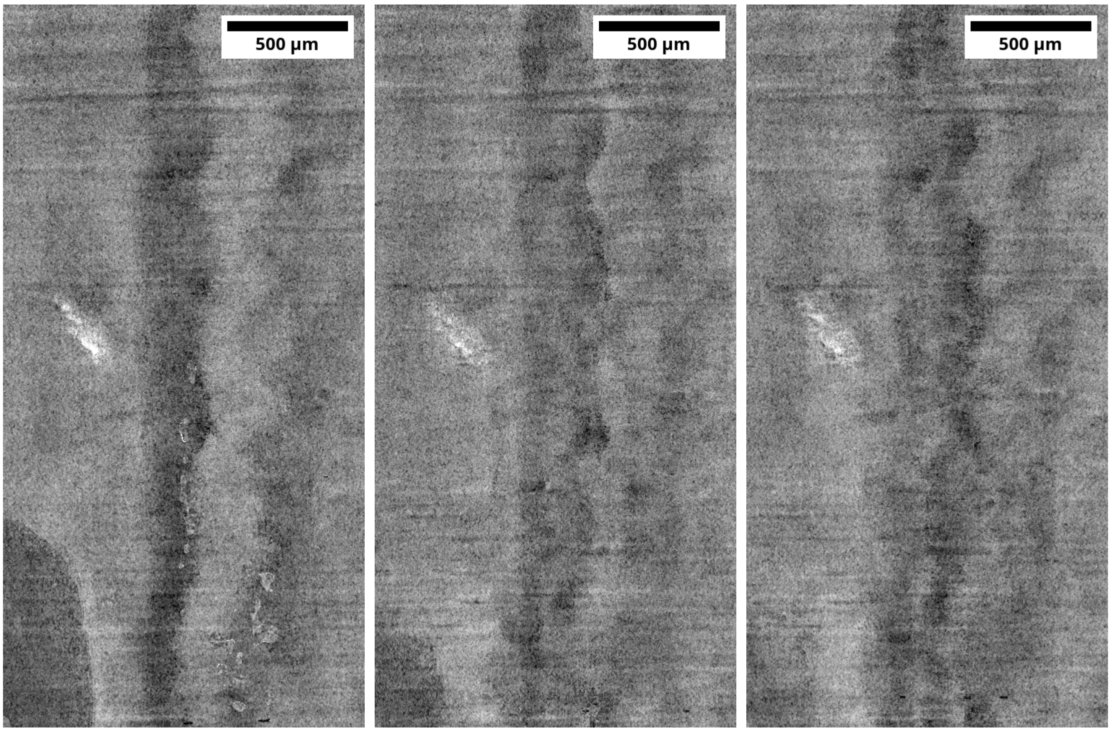

(a) BOL (b) 1st scan (c) EOL

Figure S31: >200mA/cm^2; *microporous*. The hole on the bottom left in a) is a gap between AEL and CEL due to imperfect assembly and successively closed in b,c) by CEL swelling.

5 ACL segmentation

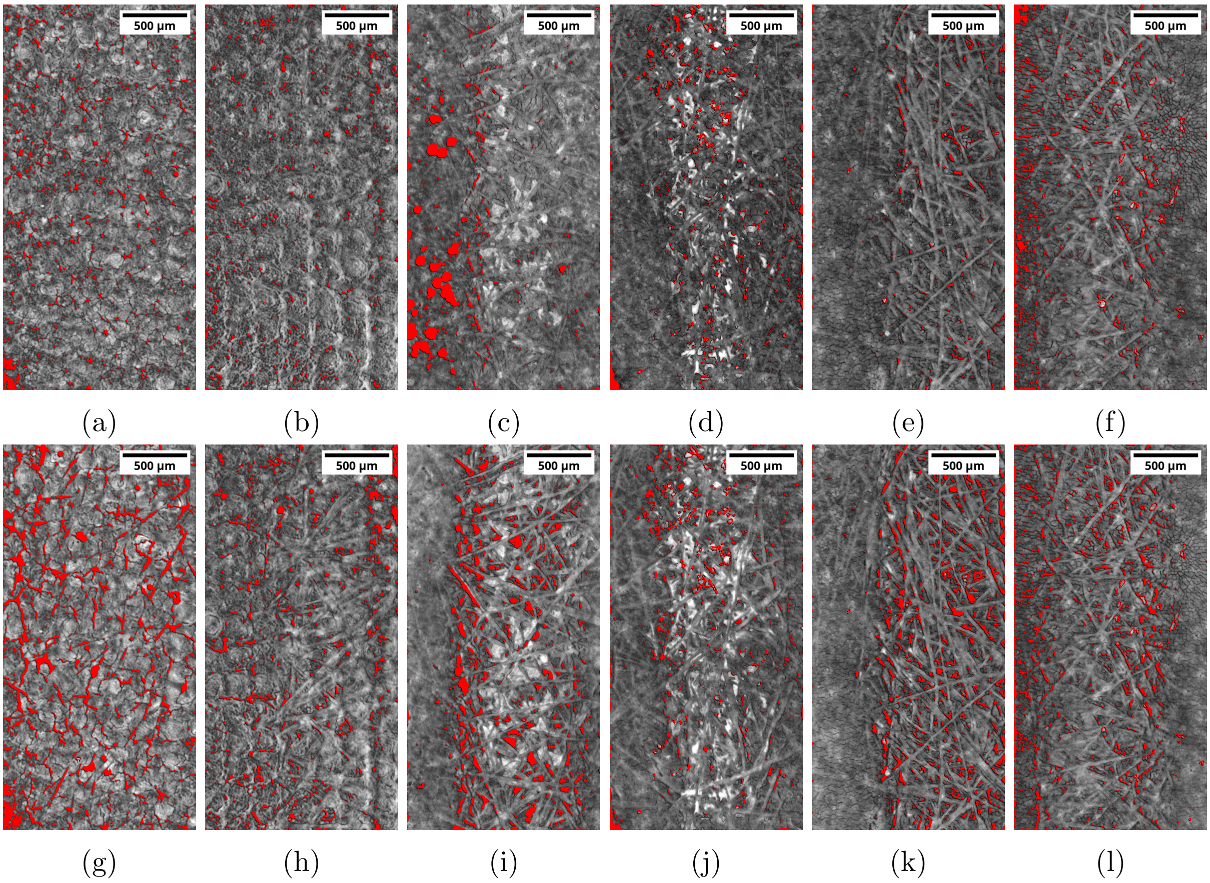

Figure S32: ACL segmentation after operation for 1h at 100mA/cm^2 for the cases a,g) *commercial* BPM with carbon GDL at anode, b,h) *commercial* BPM with Ti-PTL at anode, c,i) *custom*, d,j) *macroporous*, e,k) *mesoporous*, f,l) *microporous*. Due to "bubbly" uncorrectable segmentation errors in the left third of c), only the remaining two-thirds of the image were considered in the subsequent quantification in this particular case. Top row a-f) BOL, bottom row g-l) EOL

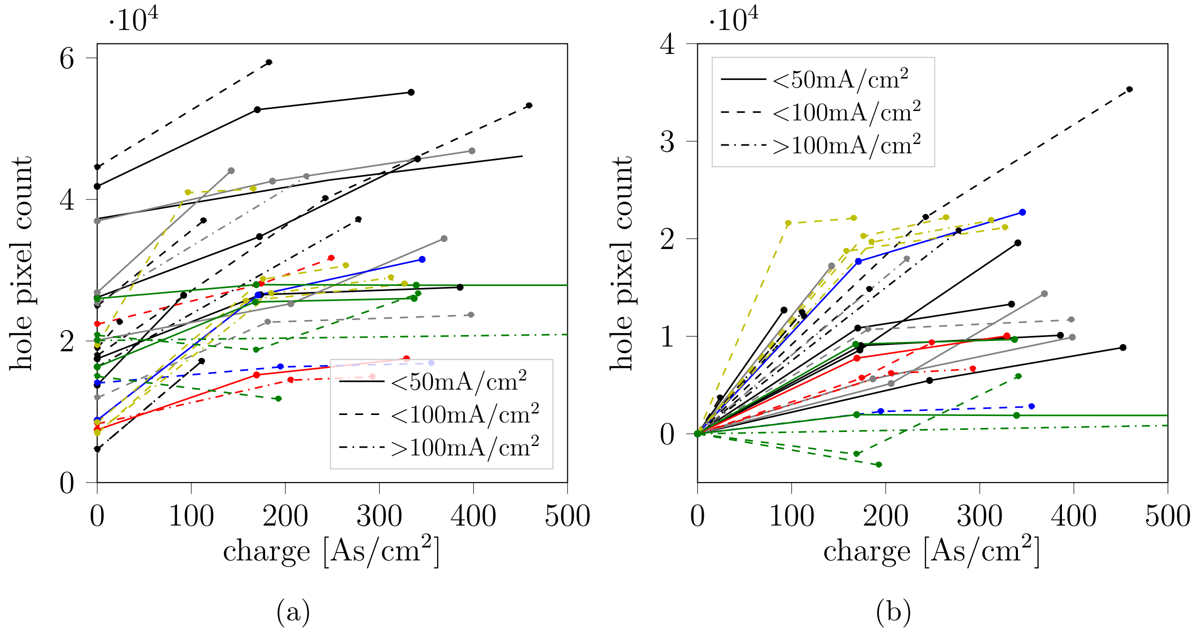

Figure S33: a) Raw relation for each sample grouped by BPM-variant and low-medium-high current density. b)Damage vs charge calibrated for the holes already present at BOL

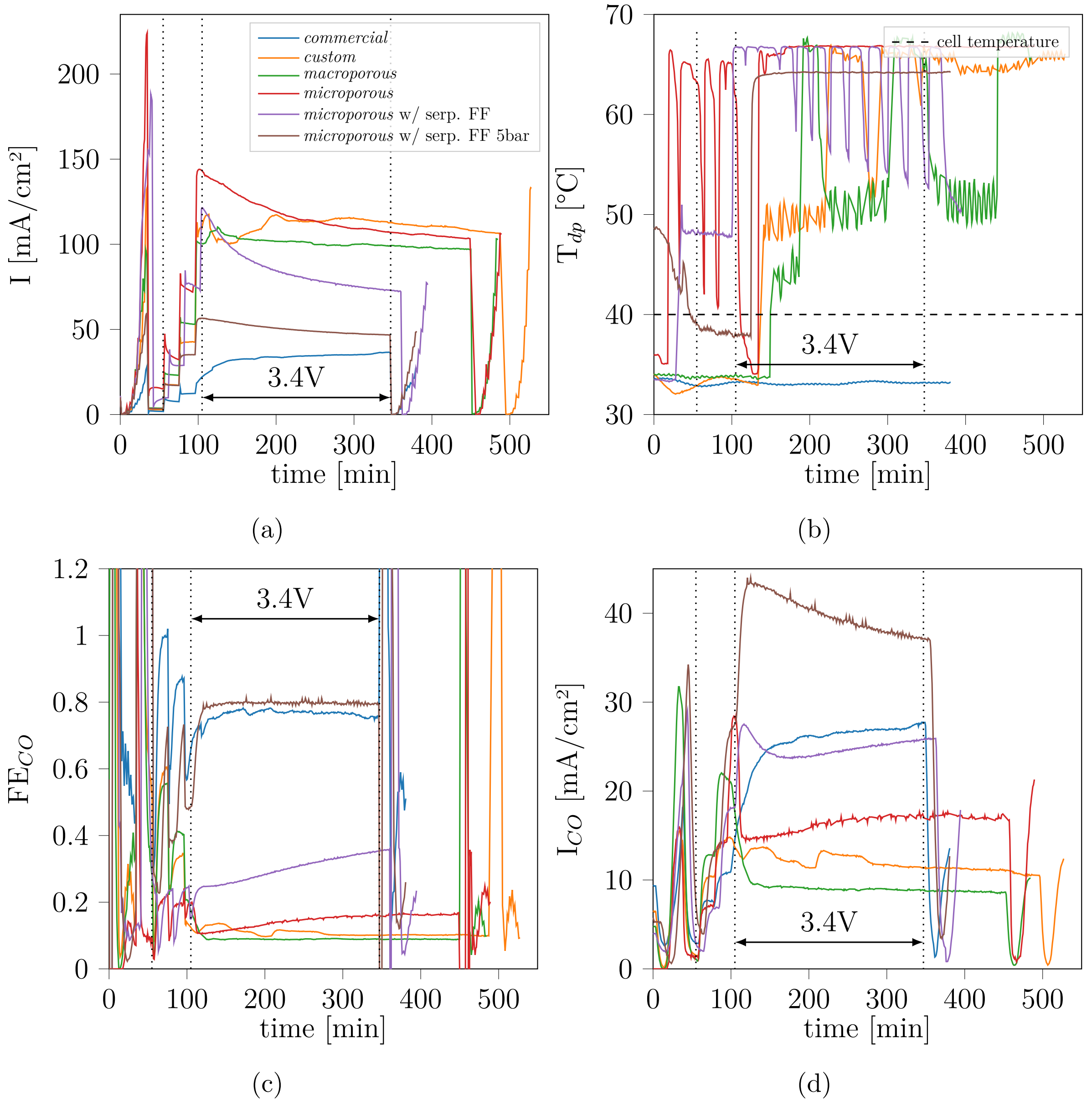

Figure S34: a) total current density over time for the entire operation including preconditioning and pseudo polarization curves, b) outlet dew point temperature, c) partial CO current density, d) CO production faradaic efficiency. The dotted lines indicate the change of potentiostatic operation. The arrow gives the period of interest with constant volatage of 3.4V.

6 Modeling interfacial membrane pressure

The parameters in a previously developed CO_2 balance model[2] can be adapted to test the influence of AEL thickness and increasing the pressure at the cathode on the CO_2 pressure at the BPM interface. We use the same reasonable material parameters (table 1) motivated in[2]. Assuming a fixed conversion of charger carriers to molecular of 0.5 (equal mix of HCO_3^- and CO_3^{2-}[2]) leads to a linear relation of CO_2 pressure at the membrane interface with applied current density. A thinner AEL decreases the slope of this relation. Reasonably assuming a minimal required pressure difference between membrane interface and surface to cause delamination and rigid counter-pressure of the anode even without pressurizing the anode, both, a thinner AEL and increasing the cathode pressure only shift this critical pressure to higher, but still attainable, current densities (figure S35). Weng *et al.*[3] predict a shift of charge carrier type from HCO_3^- and CO_3^{2-} to OH^- for increasing current density. Mitigation of membrane delamination for low current densities might therefore be sufficient as no gaseous CO_2 might be produced at higher current densities. We can feed their relation in our model and observe that reasonable configurations can be achieved where CO_2 over-pressure at the membrane can be avoided (figure S36). However, differential CO_2 pressure also increases diffusive cross-over to the anode. The model results need to be taken with care as it assumes an established equilibrium, the parameters are material dependent and especially the charge carrier compositions need to be confirmed experimentally. However, recent works with stable FB-BPM CO_2 electrolyzers[4, 5] might fall into still safe combinations of AEL thickness and operating conditions, but could be affected by membrane delamination at higher current density.

Table 1: Parameters employed in the diffusive model. T=318K (45°C), 100% relative humidity of CO_2 gas stream. Thicknesses Δ are estimated based on the imaging results.

name	ij	D^0 $[m^2/s]$	ϵ	τ	Δ $[\mu m]$	comment
CCL	23	1.06 10^{-5}	0.5	1	8	self-diffusion of CO_2 in porous, water free catalyst layer. ϵ and τ reasonable assumption based on[6].
AEL	12	3 10^{-9}	0.36	1.5	variable	D^0 diffusivity of CO_2 in water[?, ?], ϵ water volume fraction of hydrated membrane estimated from in-house gravimetric tests, τ assumption for well connected water domains
CEL	14	3 10^{-9}	0.36	1.5	60	ref. AEL
ACL	45	3 10^{-9}	0.39	2.8	10	D^0 diffusivity of CO_2 in water[?], ϵ and τ from nano-tomography[6]

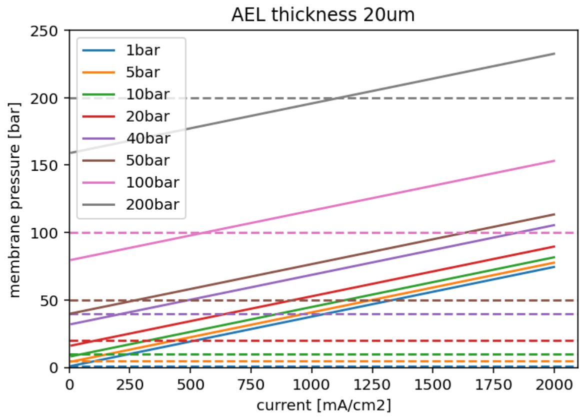

(a)

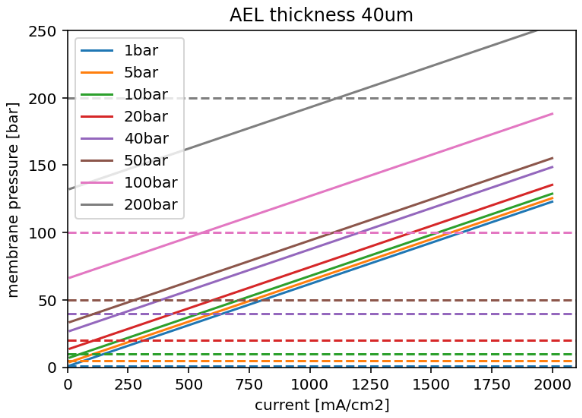

(b)

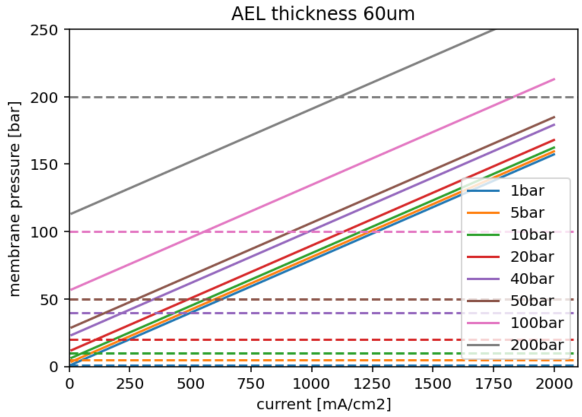

(c)

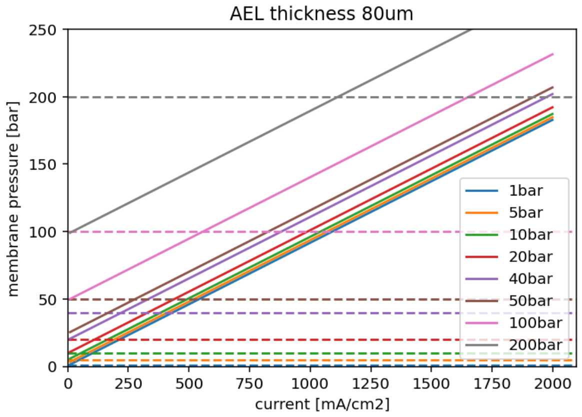

(d)

Figure S35: Modeled CO_2 pressure at membrane interface for different AEL thickness (a-d) and CO_2 pressure at cathode for a fixed conversion of charge carrier to CO_2 of 0.5. Surpassing the corresponding dashed line corresponds to CO_2 over-pressure within the BPM.

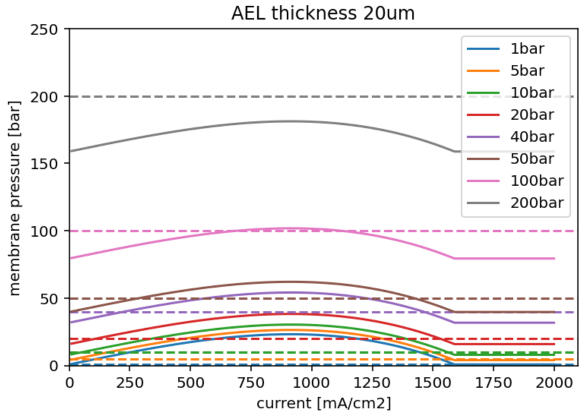

(a)

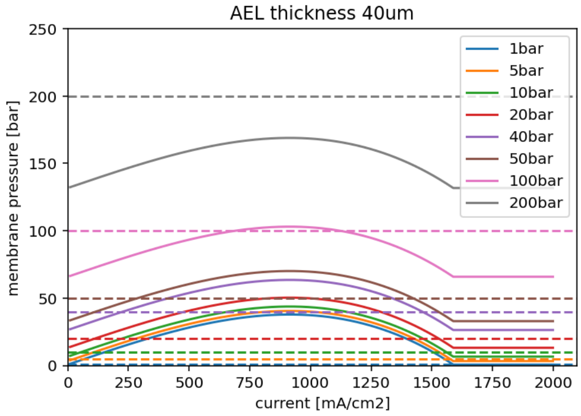

(b)

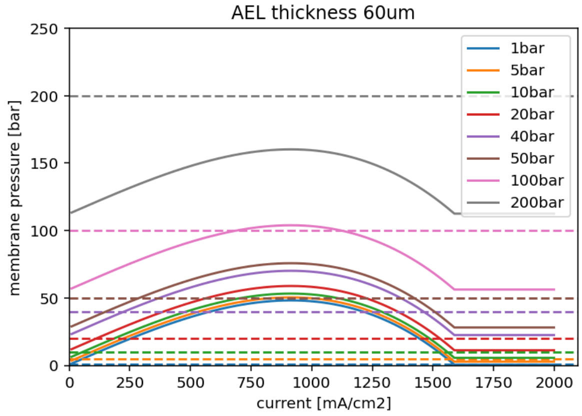

(c)

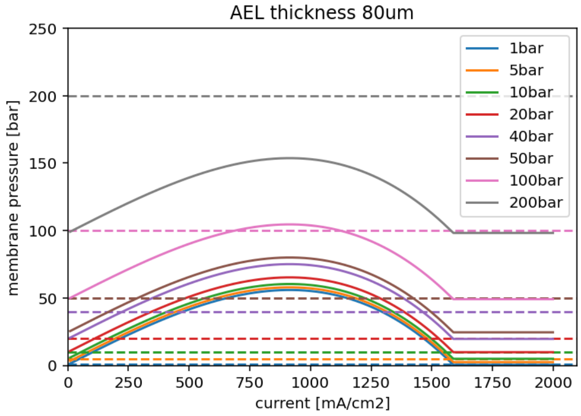

(d)

Figure S36: Modeled CO_2 pressure at membrane interface for different AEL thickness (a-d) and CO_2 pressure at cathode with variable conversion of charge carriers to CO_2 based on[3]. Surpassing the corresponding dashed line corresponds to CO_2 over-pressure within the BPM.

References

[1] I. Arganda-Carreras, V. Kaynig, C. Rueden, K.W. Eliceiri, J. Schindelin, A. Cardona, H. Sebastian Seung, Bioinformatics **33**(15), 2424 (2017). DOI 10.1093/bioinformatics/btx180

[2] R. Fischer, M.A. Dessiex, F. Marone, F.N. Büchi, ACS Applied Energy Materials **7**(9), 3590 (2024). DOI 10.1021/acsaem.3c02882

[3] L.C. Weng, A.T. Bell, A.Z. Weber, Energy & Environmental Science **12**(6), 1950 (2019). DOI 10.1039/c9ee00909d

[4] M. Heßelmann, J.K. Lee, S. Chae, A. Tricker, R.G. Keller, M. Wessling, J. Su, D. Kushner, A.Z. Weber, X. Peng, ACS Applied Materials & Interfaces **16**(19), 24649 (2024). DOI 10.1021/acsami.4c02799. URL `https://pubs.acs.org/doi/10.1021/acsami.4c02799`

[5] S. Heuser, L. Hoof, K. Pellumbi, J.N. Oberndorf, L. Krämer, D. Blaudszun, K.J. Puring, M. Prokein, N. Mölders, A. Kilzer, M. Petermann, U.P. Apfel, Chem Catalysis p. 101393 (2025). DOI 10.1016/j.checat.2025.101393. URL `https://linkinghub.elsevier.com/retrieve/pii/S2667109325001319`

[6] S. De Angelis, T. Schuler, M. Sabharwal, M. Holler, M. Guizar-Sicairos, E. Muller, F.N. Büchi, Sci Rep **13**(1), 4280 (2023). DOI 10.1038/s41598-023-30960-x
